# Brown adipose TRX2 deficiency activates mtDNA-NLRP3 to impair thermogenesis and protect against diet-induced insulin resistance

**DOI:** 10.1172/JCI148852

**Published:** 2022-05-02

**Authors:** Yanrui Huang, Jenny H. Zhou, Haifeng Zhang, Alberto Canfran-Duque, Abhishek K. Singh, Rachel J. Perry, Gerald I. Shulman, Carlos Fernandez-Hernando, Wang Min

**Affiliations:** 1 Interdepartmental Program in Vascular Biology and Therapeutics, Department of Pathology,; 2 Interdepartmental Program in Vascular Biology and Therapeutics, Department of Comparative Medicine, and; 3 Department of Internal Medicine, Yale School of Medicine, New Haven, Connecticut, USA.

**Keywords:** Inflammation, Metabolism, Adipose tissue, Innate immunity, Mitochondria

## Abstract

Brown adipose tissue (BAT), a crucial heat-generating organ, regulates whole-body energy metabolism by mediating thermogenesis. BAT inflammation is implicated in the pathogenesis of mitochondrial dysfunction and impaired thermogenesis. However, the link between BAT inflammation and systematic metabolism remains unclear. Herein, we use mice with BAT deficiency of thioredoxin-2 (TRX2), a protein that scavenges mitochondrial reactive oxygen species (ROS), to evaluate the impact of BAT inflammation on metabolism and thermogenesis and its underlying mechanism. Our results show that BAT-specific TRX2 ablation improves systematic metabolic performance via enhancing lipid uptake, which protects mice from diet-induced obesity, hypertriglyceridemia, and insulin resistance. TRX2 deficiency impairs adaptive thermogenesis by suppressing fatty acid oxidation. Mechanistically, loss of TRX2 induces excessive mitochondrial ROS, mitochondrial integrity disruption, and cytosolic release of mitochondrial DNA, which in turn activate aberrant innate immune responses in BAT, including the cGAS/STING and the NLRP3 inflammasome pathways. We identify NLRP3 as a key converging point, as its inhibition reverses both the thermogenesis defect and the metabolic benefits seen under nutrient overload in BAT-specific *Trx2*-deficient mice. In conclusion, we identify TRX2 as a critical hub integrating oxidative stress, inflammation, and lipid metabolism in BAT, uncovering an adaptive mechanism underlying the link between BAT inflammation and systematic metabolism.

## Introduction

Obesity related to overnutrition is driven by energy intake and energy expenditure (EE) imbalance. White adipose tissue (WAT), having small numbers of mitochondria, plays a major role in energy storage, but does not contribute substantially to EE (1). On the other hand, brown adipose tissue (BAT), characterized by densely packed mitochondria, is a major contributor to EE that produces heat via adaptive thermogenesis to maintain body temperature (2–4). Yet the role of BAT in maintaining whole-body metabolism homeostasis under metabolic stress remains controversial. Some studies suggest that BAT activation promotes adaptive thermogenesis and increases EE, therefore protecting against obesity, insulin resistance, and hyperlipidemia (5–7), whereas other studies indicate that the defective adaptive thermogenesis in BAT is an adaptation to metabolic stress with metabolic benefits (8, 9). Since substantial amounts of metabolically active BAT have been reported in human adults (10–13), understanding the metabolic role of BAT activity under metabolic stress could lead to new therapies for obesity and diabetes.

An unexplored aspect of BAT function involves the regulation of mitochondrial-associated inflammation in response to metabolic stress. In response to cold, BAT activates a β3-adrenergic receptor–regulated thermogenic program in which fatty acids (FAs) are transported into mitochondria for FA oxidation and uncoupling respiration to dissipate energy (14, 15). The mitochondrion, critical for energy production, can also integrate signals and initiate cellular actions under various conditions (16–18). For example, stress-induced mitochondrial dysfunction and sterile inflammation are interwoven, as mitochondria can activate aberrant innate immune responses (19, 20). Mitochondria influence the innate immune system by releasing numerous proinflammatory signals, such as reactive oxygen species (ROS), mitochondrial DNA (mtDNA), and Ca^2+^, which activate inflammasome and cyclic GMP/AMP synthase (cGAS)/cGAMP/stimulator of IFN genes (STING) pathways (21–23). Mitochondrial ROS (mtROS), one of the most critical proinflammatory signals, could be scavenged by the mitochondrial thioredoxin antioxidant system, which compresses thioredoxin-2 (TRX2), TRX2 reductase (TRXR2), and TRX2-dependent peroxiredoxin 3 (PRX3) (24). In support of a critical role for TRX2 in regulating inflammation, TRX2 suppresses the activation of the inflammatory transcription factor NF-κB by reducing oxidation of Cys disulfide (Trx-S2; ref. 25). On the other hand, inflammation activation in BAT disrupted mitochondria integrity, further leading to impaired thermogenic activity (26, 27). These prior studies indicate a crucial role of BAT inflammation in adaptive thermogenesis. However, the role of BAT mitochondrial inflammation in systematic metabolism under metabolic stress is mostly unexplored.

BAT inflammation might be involved in regulating systematic metabolism under metabolic stress, evidenced by BAT mitochondrial damage, cold intolerance, and metabolic abnormalities of *ob/ob* mice (28). Moreover, our recent study about mice with whole fat–specific deficiency of *Trx2* using adiponectin-Cre (*Trx2^ADKO^*) indicates that these mice developed hyperglycemia, hepatic insulin resistance, and hepatic steatosis while showing mitochondria abnormalities in BAT (29); however, the role of BAT inflammation in whole-body metabolism could not be determined, as the previous studies used systematic inflammation models.

To address the current question, we generated brown adipocyte–specific TRX2-KO mice (*Trx2^BATKO^* mice). Our study shows that BAT-specific TRX2 deficiency stimulates the innate immune response via unchecked mtROS production, triggers NACHT, LRR, and PYD domain–containing protein 3 (NLRP3) inflammasome activation and cGAS/cGAMP/STING signaling, and ultimately exacerbates sterile inflammation. Additionally, loss of TRX2 in BAT impairs thermogenesis by disrupting mitochondrial integrity, thus suppressing FA oxidation. In contrast to the ablation of *Trx2* in both WAT and BAT in *Trx2^ADKO^*, we observed metabolic benefits after the ablation of TRX2 in BAT, including protection from diet-induced obesity, hypertriglyceridemia, and insulin resistance. The current study has uncovered an adaptive mechanism linking BAT inflammation and systematic metabolism.

## Results

### BAT-specific TRX2 deficiency protects mice from diet-induced obesity, insulin resistance, and hypertriglyceridemia.

Our previous study suggested that whole fat–specific TRX2 deficiency impaired whole-body lipid and glucose metabolism (29). We determined the expression of TRX2 in various adipose tissues. TRX2 expression was much higher in BAT than in inguinal WAT (ingWAT) and epididymal WAT (eWAT) (Supplemental Figure 1A; supplemental material available online with this article; https://doi.org/10.1172/JCI148852DS1). Uncoupling protein 1 (UCP1), a brown adipose marker, was expressed in BAT, but not in ingWAT or eWAT at room temperature (RT), which is consistent with previous independent studies (30). Therefore, we generated BAT-specific KO mice (*Trx2^BATKO^*) by crossing *Ucp1-Cre* with *Trx2^fl/fl^* mice. Western blot analysis confirmed the specific depletion of TRX2 in BAT (~15-fold), but not in WAT, of *Trx2^BATKO^* mice (Supplemental Figure 1B). A significant decrease in TRX2 abundance in isolated mature brown adipocytes, but not in the stromal vascular fraction from BAT, further validated *Ucp1-Cre* specificity (Supplemental Figure 1C). *Trx2^BATKO^* mice exhibited body weights and food intake similar to those of WT mice (Supplemental Figure 1, D and E), yet showed reduced total fat composition (Supplemental Figure 1F), which was mostly due to a decrease (~30%) in weight of ingWAT. In contrast, eWAT and retroperitoneal WAT (rWAT) fat masses were not altered (Supplemental Figure 1G). Surprisingly, *Trx2^BATKO^* mice had higher total BAT mass, including interscapular BAT (iBAT), subscapular BAT (sBAT), and cervical BAT (cBAT) fat masses (Supplemental Figure 1G). Enlarged iBAT was attributed to steatotic hypertrophy, as we observed in the accumulation of larger and more unilocular lipid droplets (LDs) in adipocytes from *Trx2^BATKO^* mice compared with those in WT (Supplemental Figure 1H). However, as the total amount of DNA per iBAT showed no significant difference (Supplemental Figure 1I), hyperplasia likely did not contribute to increased BAT fat mass in *Trx2^BATKO^* mice. Triglyceride (TGs) measurements indicated increased TG contents in iBAT and decreased TG within ingWAT in *Trx2^BATKO^* mice (Supplemental Figure 1J). Further histologic examination of *Trx2^BATKO^* mice revealed more multilocular adipocytes in ingWAT, but not in eWAT or livers (Supplemental Figure 1K). Fasting plasma glucose levels (Supplemental Figure 1L) and insulin levels (Supplemental Figure 1M) were not altered in *Trx2^BATKO^* mice under normal chow diet (NCD), and neither glucose tolerance nor insulin sensitivity differed between cohorts (Supplemental Figure 1, N and O). Furthermore, we observed no differences in circulating LDL cholesterol (LDL-C), total cholesterol (TC), HDL cholesterol (HDL-C), and TG between cohorts (Supplemental Figure 1, P–S). These data suggest that TRX2 deficiency in BAT induces BAT steatotic hypertrophy without metabolic disorder under NCD, in contrast to the diabetic and hyperlipidemic phenotype observed in *Trx2^ADKO^* mice (29).

**Figure 1:**
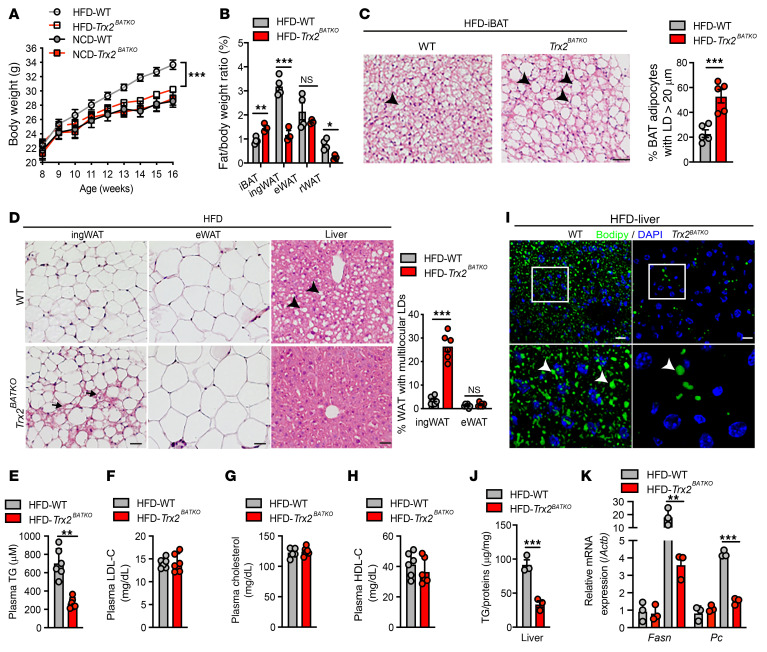
BAT-specific TRX2 deficiency protects mice from diet-induced hepatic steatosis and hypertriglyceridemia. (**A**) Growth trend of *Trx2^BATKO^* and WT mice under NCD (*n* = 6) or HFD (*n* = 10). (**B**) Ratio of adipose depots to body weight from 16-week-old *Trx2^BATKO^* (*n* = 3) and WT (*n* = 4) mice under HFD. (**C**) Representative histologic images of iBAT from HFD-fed mice. Arrowheads denote large unilocular LDs. BAT adipocytes with unilocular LD diameter of 20 μm or more were quantified. (**D**) Representative histologic images of adipose tissues and liver from *Trx2^BATKO^* and WT mice under HFD. Arrows denote multilocular LDs in WAT, while arrowheads denote LDs in the liver. Adipocytes with multilocular LDs (%) are quantified. *n* = 6. (**E**–**H**). Lipid profile, including plasma TG (**E**), LDL-C (**F**), TC (**G**), and HDL-C (**H**) levels from 16-week-old WT and *Trx2^BATKO^* mice under HFD (*n* = 6). (**I**) Representative BODIPY staining images of liver from mice under HFD. White box denotes magnified areas. Arrowheads denote LDs in the liver. (**J**) TG contents of liver from HFD-fed mice (*n* = 3). (**K**) Expression of lipid metabolic–related genes in livers from HFD-treated mice (*n* = 3). Quantitative data are presented as mean ± SEM. *P < 0.05; **P < 0.01; ***P < 0.001. Significance was assessed by 2-way ANOVA with Bonferroni’s post hoc tests (**A**) and 2-tailed Student’s *t* test (**B**–**D**, **J**, and **K**). Scale bars: 100 μm (**C** and **D**); 25 μm (**I**). Original magnification for higher magnification images, ×600 (**I**).

To determine whether the absence of TRX2 in BAT affects overnutrition-induced metabolic disorder, we fed 8-week-old mice a high-fat diet (HFD) (45% fat kcal) for 8 weeks. HFD-fed *Trx2^BATKO^* mice gained weight more slowly compared with WT littermates (Figure 1A). Consistent with the lower body weight, we found significantly lower fat masses of ingWAT (~65%) and rWAT (~70%), indicating reduced WAT adiposity in HFD-fed *Trx2^BATKO^* mice (Figure 1B). Furthermore, the white fat–appearing morphology of BAT in *Trx2^BATKO^* mice showed an increase in unilocular LDs (Figure 1C). Interestingly, the ingWAT from *Trx2^BATKO^* mice continued to contain multilocular LDs (Figure 1D with quantification of percentage of WAT with multilocular LDs).

**Figure 2:**
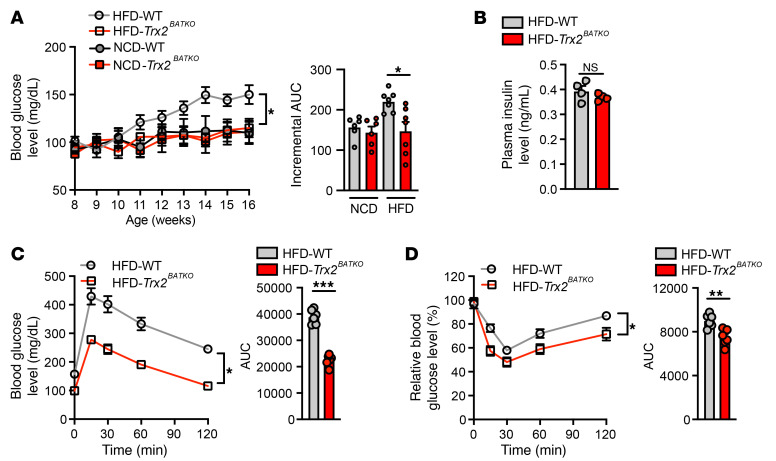
BAT-specific TRX2 deficiency protects mice from diet-induced insulin resistance and hypertriglyceridemia. (**A**) Fasting blood glucose levels of mice under NCD or HFD (*n* = 6) and quantification by incremental AUC. (**B**) Fasting plasma insulin levels of HFD-fed mice (*n* = 4). (**C**) GTT of HFD-treated mice (*n* = 6) and quantification. (**D**) Insulin tolerance test of HFD-treated mice (*n* = 6). Quantitative data are presented as mean ± SEM. *P < 0.05; **P < 0.01; ***P < 0.001. Significance was assessed by 2-way ANOVA with Bonferroni’s post hoc tests (**A**, **C**, and **D**) and 2-tailed Student’s *t* test (**B**).

To evaluate the effect of TRX2 deficiency on lipid metabolism, we assessed circulating lipids in mice fed with HFD. Remarkably, we found a significant reduction of TG in HFD-fed *Trx2^BATKO^* mice as compared with WT mice (Figure 1E), while cholesterol distribution in different lipoprotein fractions was similar between both groups of mice (Figure 1, F–H). Livers from *Trx2^BATKO^* mice exhibited less steatosis compared with those of WT mice under HFD, as indicated by reduced LD, neutral lipid accumulation, and TG content compared with WT mice (Figure 1, D, I, and J). We further examined hepatic genes involved in regulation of lipid metabolism. Consistent with reduced hepatic steatosis observed in HFD-fed *Trx2^BATKO^* mice, we found a marked decrease in FA synthase (*Fasn*) expression (~80% lower compared with WT mice). The livers of HFD-fed *Trx2^BATKO^* mice also showed an approximately 80% reduction of pyruvate carboxylase (*Pc*) expression, a gene encoding the first enzyme of gluconeogenesis, suggesting a decreased FA flux into the liver, which was in line with the observed lower serum TG level (Figure 1K). Thus, these findings suggest that overnutrition induced hepatic steatosis and hypertriglyceridemia in WT, but not in *Trx2^BATKO^*, mice. *Trx2^BATKO^* mice were resistant to diet-induced hyperglycemia (Figure 2A with incremental AUC) despite plasma insulin levels similar to those of WT mice (Figure 2B). In addition, relative to WT controls, HFD-fed *Trx2^BATKO^* mice had improved glucose tolerance (Figure 2C) and increased insulin sensitivity (Figure 2D).

Taken together, these results indicate that TRX2 deficiency in BAT protects mice from diet-induced obesity, insulin resistance, and hypertriglyceridemia. This sharply contrasts with *Trx2^ADKO^* mice, in which HFD promotes insulin resistance and hepatic steatosis by dysregulating lipid and glucose metabolism (29).

### Lack of TRX2 in BAT promotes lipid uptake.

**Figure 3:**
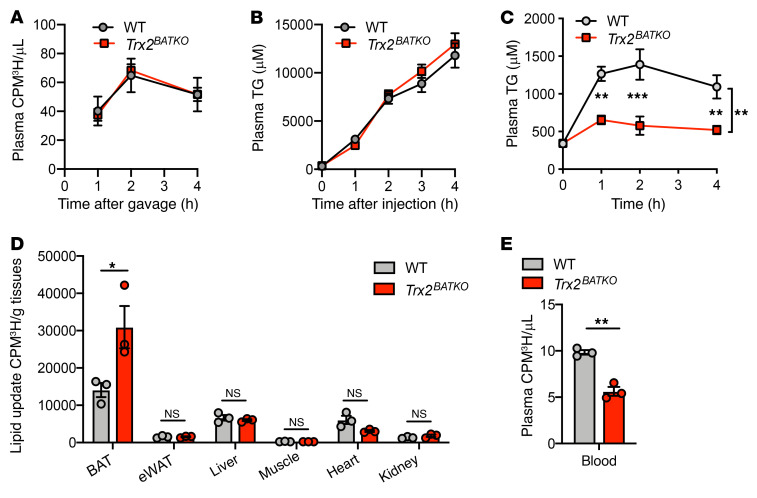
TRX2 loss enhances lipid uptake in BAT. (**A**) Intestine lipid absorption capacity of 16-week-old WT and *Trx2^BATKO^* mice by detecting plasma radioactivity under oral gavage of [^3^H]-labeled triolein along with poloxamer 407 injection (*n* = 5). CPM, counts per minute. (**B**) Hepatic VLDL production detection by measuring plasma TG levels from WT and *Trx2^BATKO^* mice under overnight fasting and treated with LPL inhibitor poloxamer 407 to inhibit catabolism (*n* = 4). (**C**) Oral lipid tolerance test performed on mice to determine circulating TG clearance (*n* = 5). (**D**–**E**) Exogenous lipid uptake in tissues determined by detecting radioactivity after oral gavage of [^3^H]-labeled triolein of WT and *Trx2^BATKO^* mice (*n* = 3). Plasma radioactivity of WT and *Trx2^BATKO^* mice are shown in **E**. Quantitative data are presented as mean ± SEM. *P < 0.05; **P < 0.01; ***P < 0.001. Significance was assessed by 2-way ANOVA with Bonferroni’s post hoc tests (**A**–**C**) and 2-tailed Student’s *t* test (**D** and **E**).

*Trx2^BATKO^* mice have lower TG profiles than control littermates under HFD. The level of plasma TG is determined mainly by 3 factors: intestinal absorption, hepatic TG secretion, and lipid uptake by peripheral tissues. Postprandial lipids, mainly TGs, are first absorbed by the small intestine and then secreted in plasma in the form of chylomicrons (31). Intestinal absorption was likely not altered, as an oral fat-tolerance test with [^3^H]-triolein in the presence of poloxamer 407, a lipoprotein lipase (LPL) inhibitor, revealed no significant difference in plasma [^3^H]-radioactivity after inhibiting lipoprotein catabolism (Figure 3A). Next, we investigated whether impaired secretion of hepatic very low-density lipoprotein (VLDL) contributed to the decrease of plasma TG levels in *Trx2^BATKO^* mice. We observed no difference between cohorts in fasting plasma TG levels after injection with poloxamer 407 (Figure 3B). Therefore, neither intestinal absorption nor hepatic secretion contributed to reduced plasma TG levels in *Trx2^BATKO^* mice.

We hypothesized that hypotriglyceridemia in *Trx2^BATKO^* mice was due to enhanced lipid uptake in tissues. A fat-tolerance test revealed a significantly lower level of plasma TG after olive oil gavage in *Trx2^BATKO^* mice (Figure 3C). Circulating TGs are delivered to peripheral tissues in the form of triglyceride-rich lipoproteins (TRLs), which are rapidly hydrolyzed into glycerol and FAs by LPL (32). To further investigate which tissue contributed to accelerated TG clearance, mice were given olive oil gavage with [^3^H]-triolein. As previously reported (7, 33), BAT had significantly higher lipid uptake activity compared with other tissues, including eWAT (Figure 3D). Furthermore, TRX2 loss promoted lipid uptake by approximately 2-fold in BAT, but not in other tissues (Figure 3D), corresponding to the approximately 50% decrease of plasma [^3^H]-triolein radioactivity (Figure 3E). These data are consistent with a reduced TG accumulation observed in the BAT of *Trx2^BATKO^* mice. Overall, TRX2 loss in BAT enhances circulating TG clearance and improves whole-body lipid metabolism by increasing BAT lipid uptake.

### TRX2 deficiency in BAT improves whole-body glucose tolerance by enhancing glucose metabolism in WAT, liver, and skeletal muscle.

Previous studies have suggested that BAT activation can improve systemic insulin resistance by activating its glucose metabolism (34, 35). To explore whether the better utilization of glucose of *Trx2^BATKO^* mice is attributed to BAT, we further determined the expression of critical genes regulating aerobic oxidation of glucose in BAT. Glucose metabolism involves various processes, including glucose uptake, glycolysis, and aerobic oxidation (Supplemental Figure 2A). In contrast to our expectation, KO of TRX2 in BAT reduced the expression of glucose transporter type 4 (Glut4), which encoded the critical protein that transports glucose into brown adipocytes, suggesting a restraint of glucose uptake. Moreover, BAT of *Trx2^BATKO^* mice had a significantly lower expression of glucokinase (Gck), the first rate-limiting enzyme during glycolysis, and pyruvate dehydrogenase (Pdh1), the enzyme that converts pyruvate into acetyl-CoA to enter the TCA cycle (Supplemental Figure 2B). Furthermore, a HFD could not restore the expression of these genes in *Trx2^BATKO^* mice, indicating that glucose metabolization is retrained (Supplemental Figure 2C). Indeed, in vitro glycolysis assay demonstrated a dramatic reduction of glycolysis efficiency in TRX2-deficient brown adipocytes (Supplemental Figure 2D). These data suggest that TRX2 deficiency in BAT suppressed its utilization of glucose, in line with the impaired glucose metabolism observed in eWAT of *Trx2^ADKO^* mice (29).

Since skeletal muscle, liver, and ingWAT are 3 organs crucial for glucose metabolism and targets of insulin (36), we determined the expression of genes involving glucose uptake and glycolysis in these tissues. GLUT, the key enzyme of glucose uptake, has different isotypes. GLUT2 is responsible for glucose uptake in liver, while GLUT4 is attributed to glucose uptake in ingWAT and skeletal muscle. *Glut2* expression of liver and *Glut4* expression of ingWAT were significantly downregulated in *Trx2^BATKO^* mice under chow diet, while *Glut4* expression of skeletal muscle showed no difference. However, the *Gck* and *Pdh1* of all 3 tissues were upregulated in *Trx2^BATKO^* mice, suggesting an augmented activity of glucose utilization (Supplemental Figure 2, E–H). Furthermore, under the stress of overnutrition, TRX2 deficiency in BAT had raised the expression of genes related to glucose uptake and glycolysis in all 3 tissues (Supplemental Figure 2, I–L). These data suggest that the better insulin sensitivity of *Trx2^BATKO^* mice may be attributed to the enhanced glucose metabolization from muscle, liver, and ingWAT. Overall, our data suggest that the loss of TRX2 in BAT ameliorates systematic glucose metabolism, which is mainly attributed to the compensatory activation of insulin-targeted tissues other than BAT.

### Absence of TRX2 in BAT impairs cold-induced thermogenesis via suppressing FA oxidation.

**Figure 4:**
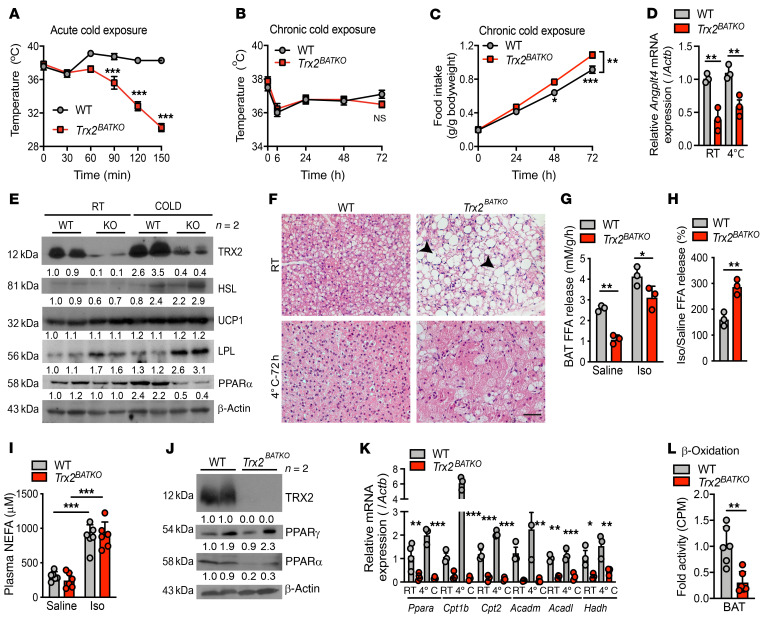
TRX2 deficiency in BAT impairs thermogenesis. (**A**) Intrarectal temperature of 16-week-old WT and *Trx2^BATKO^* mice during acute cold intolerance test in the absence of food (*n* = 6). (**B**–**F**) WT and *Trx2^BATKO^* mice after 3 days of cold exposure. (**B**) Intrarectal temperature (*n* = 6). (**C**) Food intake during cold exposure. (**D**) Angptl4 mRNA expression in iBAT (*n* = 3). (**E**) Western blots of indicated proteins in iBAT. Relative protein levels are presented as fold changes by taking WT as 1.0. *n* = 2. (**F**) Histologic iBAT images. Arrowheads denote large LDs. (**G** and **H**) Ex vivo lipolysis assay detecting FFA levels (**G**) and FFA release efficiency (**H**) of isolated iBAT treated with saline or ISO (*n* = 3). (**I**) In vivo lipolysis assay detecting plasma NEFA (*n* = 6). (**J**) PPARα and PPARγ protein expression in iBAT. Relative protein levels are presented by taking saline as 1.0. *n* = 2 mice. (**K**) Relative mRNA expression of *Ppara* (*n* = 4) and its target genes (*n* = 3) in iBAT. (**L**) Ex vivo FA oxidation of iBAT (*n* = 6). Quantitative data are presented as mean ± SEM. *P < 0.05; **P < 0.01; ***P < 0.001. Two-way ANOVA with Bonferroni’s post hoc tests (**A**–**C**), 1-way ANOVA followed by Tukey’s post hoc test (**G** and **I**), and 2-tailed Student’s *t* test (**D**, **H**, **K**, and **L**). Scale bar: 100 μm (**F**).

BAT is essential for dissipating energy to heat during cold exposure in rodents and humans (37, 38). Lipid uptake is crucial during cold-induced thermogenesis, as BAT uptake of TG dramatically accelerates under cold exposure (7). Since we detected enhanced BAT lipid uptake in *Trx2^BATKO^* mice, we explored their thermogenic ability by placing 16-week-old WT and *Trx2^BATKO^* mice at 4°C. Contrary to our hypothesis, *Trx2^BATKO^* mice were cold intolerant, reaching critical hypothermia (30°C) within 3 hours (Figure 4A). We further challenged WT and *Trx2^BATKO^* mice at 4°C for 72 hours. Surprisingly, the cold-exposed ad libitum–fed *Trx2^BATKO^* mice maintained normal body temperature with significantly increased food intake compared with WT mice (Figure 4, B and C), indicating that hypothermia in acute cold-exposed fasted *Trx2^BATKO^* mice could be reversed in the presence of sufficient exogenous substrate supply. Several independent studies found that stimulated BAT activity is mainly related to cold-induced thermogenesis instead of diet-induced thermogenesis (39–41). Thus, our findings suggested an impaired BAT activation under cold exposure upon *Trx2* deficiency. The enhanced lipid uptake of *Trx2^BATKO^* mice we observed at RT was continued under cold exposure, evidenced by the upregulation of LPL expression and the downregulated expression of its inhibitor, angiopoietin-like 4 (Angptl4) (Figure 4, D and E). Therefore, our findings suggest that TRX2 might influence BAT lipid metabolism pathways other than lipid intake to regulate cold-induced thermogenesis.

Though controversial, intracellular lipolysis and FA oxidation in brown adipocytes are generally considered necessary in cold-induced thermogenesis. Though BAT of *Trx2^BATKO^* mice showed a lower protein expression of hormone-sensitive lipase (HSL) at RT, we observed comparable increases of HSL expression in WT and *Trx2^BATKO^* mice after cold exposure (Figure 4D). Consistently, histologic images showed a reversed morphology of smaller LDs after chronic cold exposure in BAT of *Trx2^BATKO^* mice compared with RT (Figure 4F), indicating enhanced lipolysis in BAT. Furthermore, ex vivo analysis of lipolysis in BAT demonstrated that even though the ability to release free FAs (FFAs) in BAT of *Trx2^BATKO^* mice was suppressed in basal conditions, it could recover in response to isoproterenol (ISO), a β-adrenergic receptor agonist (Figure 4, G and H). These findings suggest that suppressed lipolytic ability in BAT from *Trx2^BATKO^* mice can be activated by cold stimulation. Indeed, in vivo lipolysis experiments showed no difference between WT and *Trx2^BATKO^* mice in ISO-stimulated circulating FAs (Figure 4I). Altogether, TRX2 ablation had little effect on brown adipocyte intracellular lipolysis. Of note, serum FFAs are dramatically increased in *Trx2^ADKO^* mice due to impaired suppression of WAT lipolysis, leading to ectopic lipid deposition in liver, where it induces hepatic lipogenesis and gluconeogenesis (29). Therefore, TRX2 deficiency in WAT and BAT affects lipolysis differently, leading to distinct outcomes in metabolic phenotypes.

To investigate the contribution of FA oxidation to defective thermogenesis, we detected the protein expression of PPARα, a critical transcriptional factor regulating FA oxidation in BAT, and found that PPARα was downregulated in *Trx2^BATKO^* mice (Figure 4J). However, the protein level of PPARγ, a transcriptional factor regulating lipogenesis, was unchanged (Figure 4J). After chronic cold exposure, *Ppara* and its downstream target mitochondrial β-oxidation genes, including carnitine palmitoyltransferase 1B (*Cpt1b*), carnitine palmitoyltransferase 2 (*Cpt2*), acyl–coenzyme A dehydrogenase medium-chain (*Acadm*), acyl–coenzyme A dehydrogenase long-chain (*Acadl*), and hydroxyacyl–coenzyme A dehydrogenase (*Hadh*), remained significantly diminished in *Trx2^BATKO^* mice (Figure 4, D and K), suggesting an inability of FA oxidation under cold induction. Indeed, ex vivo assay demonstrated retained FA oxidation in BAT of *Trx2^BATKO^* mice (Figure 4L). Together, these results indicate that impaired FA oxidation may be the primary cause of impaired thermogenesis in the absence of TRX2.

**Figure 5:**
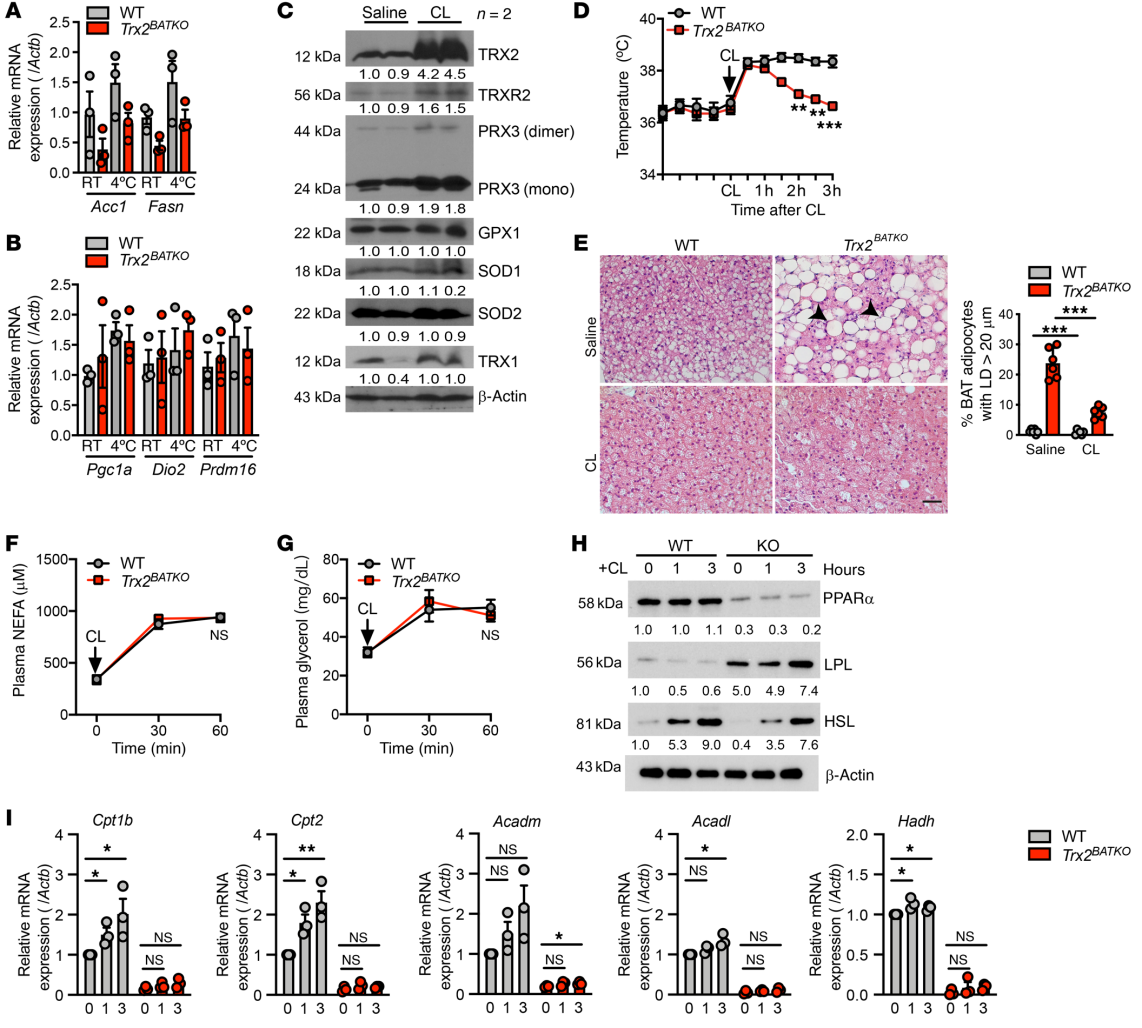
TRX2 BAT ablation suppresses FA oxidation. (**A** and **B**) Gene expression in iBAT from mice after 3 days of cold exposure (*n* = 3). (**C**–**I**) Mice treated with CL136,243 (1 mg/kg) for 3 hours. (**C**) Antioxidant protein levels in iBAT from WT mice by taking saline as 1.0 (*n* = 2). (**D**) Intrarectal temperature (*n* = 6). (**E**) Histologic images of iBAT. Arrowheads denote large LDs, with quantification in the right panel (*n* = 6). (**F** and **G**) Plasma NEFA and glycerol levels (*n* = 4). (**H**) Immunoblots of proteins in iBAT (each lane from 3 mice). (**I**) Relative mRNA expression of genes in iBAT (*n* = 3). Quantitative data are presented as mean ± SEM. *P < 0.05; **P < 0.01; ***P < 0.001. Two-tailed Student’s *t* test (**A** and **B**), and 1-way ANOVA followed by Tukey’s post hoc test (**D**, **F**, **G**, and **I**). Scale bar: 100 μm (**E**).

Additionally, acetyl–coA carboxylase 1 (*Acc1*) and *Fasn*, 2 lipogenesis-related genes, showed similar expression between cohorts at RT and after cold exposure (Figure 5A). Finally, canonical cold-induced genes, including *Ucp1*, peroxisome proliferator–activated receptor γ coactivator 1-α (*Pgc1a*), iodothyronine deiodinase 2 (*Dio2*), and PR domain containing 16 (*Prdm16*), showed expression in *Trx2^BATKO^* mice similar to that in WT mice at RT and after chronic cold exposure (Figure 5B). These findings indicate that the loss of TRX2 does not affect thermogenesis through lipogenesis or the canonical thermogenic pathway.

In addition to brown adipose thermogenesis, shivering-induced thermogenesis and cardiac muscle also contribute to maintaining body temperature during cold exposure (42). To eliminate the effect of skeletal muscle shivering on thermogenesis, we activated nonshivering thermogenesis via injecting a selective β3-adrenergic receptor agonist, CL316,243, into the mice. WT mice showed markedly enhanced TRX2, TRXR2, and TRX2-dependent PRX3 after CL316,243 treatment. Of note, PRX3 showed a significant increase in the dimer/monomer ratio, indicative of oxidative stress responses (Figure 5C). Therefore, significantly enhanced TRX2, TRXR2, and PRX3 expression after CL316,243 treatment could be a response to elevated mtROS levels. However, we observed no changes in expression of the cytosolic thiol redox proteins (Figure 5C). Combined with the cold intolerance of *Trx2^BATKO^* mice, these data indicate a crucial role of TRX2 in thermogenesis activation. Indeed, as with the cold exposure test, we found that, despite a similar temperature increase in response to CL316,243 injection, *Trx2^BATKO^* mice were unable to maintain WT body temperature within 3 hours of injection (Figure 5D). Histologic images revealed inhibition of skeletal muscle shivering induced morphologic changes similar to those of the cold exposure in BAT of *Trx2^BATKO^* mice (Figure 5E). Meanwhile, plasma levels of nonesterified FA (NEFA) or glycerol levels were similar between WT and *Trx2^BATKO^* mice (Figure 5, F and G), consistent with our in vivo lipolysis experiment using ISO.

We further determined the expression of lipid metabolism–related proteins at 1 and 3 hours after CL316,243 injection. Our data showed that HSL expression was comparably increased between cohorts after CL316,243 injection. In addition, the rise of LPL expression in BAT of *Trx2^BATKO^* mice was further elevated by CL316,243 treatment (Figure 5H). Nevertheless, the protein expression of PPARα and mRNA expression of mitochondrial β-oxidation genes remained significantly declined (Figure 5, H and I). These findings indicate that the transient increase in body temperature following CL316,243 treatment in *Trx2^BATKO^* mice was likely attributed to the activation of lipid uptake and lipolysis, which increased the substrate flux. The incapacity to maintain temperature in *Trx2^BATKO^* mice was mainly caused by suppression of the ability of FA oxidation to utilize the lipid substrates. These alternations of lipid metabolism likely weakened the response of BAT to cold-induced β3-adrenergic receptor agonists in *Trx2^BATKO^* mice.

### Loss of TRX2 in BAT contributes minimally to whole-body EE.

**Figure 6:**
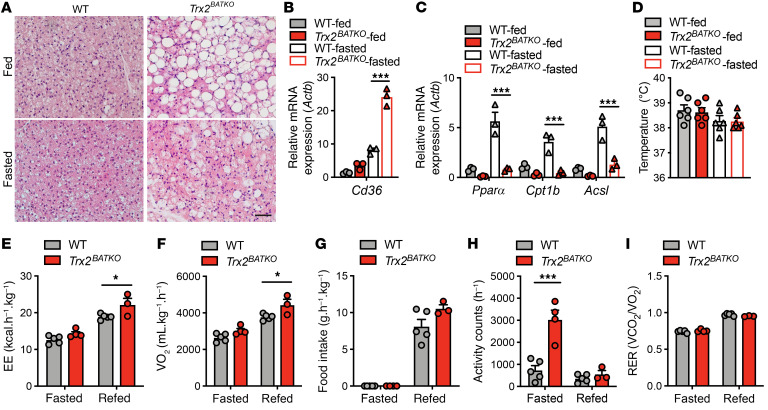
TRX2 loss in BAT contributes minimally to whole-body EE. (**A**–**D**) Mice were fed or fasted for 24 hours from 7 am. (**A**) Representative histologic images of iBAT from 16-week-old WT and *Trx2^BATKO^* mice. Three sections from each mouse (*n* = 3 mice). (**B**) *Cd36* expression in iBAT (*n* = 3). (**C**) Gene expression of FA oxidation–related genes in iBAT (*n* = 3 mice). (**D**) Intrarectal temperature (*n* = 6). (**E**–**I**). Mice were subjected to metabolic cage evaluation. Mice were individually placed in metabolic cages (CLAMs) and allowed to acclimatize for 48 hours before readings were taken. Mice were fasted for 24 hours and refed for 48 hours (*n* = 5 for WT, *n* = 4 for KO in fasted, *n* = 3 for KO in refed). EE (**E**), oxygen consumption (VO_2_) (**F**), food intake (**G**), physical activity counts (**H**), and RER (**I**) were measured. Analyses were performed in male mice. Quantitative data are presented as mean ± SEM. *P < 0.05; ***P < 0.001. Significance was assessed by 2-tailed Student’s *t* test. Scale bar: 100 μm (**A**).

In the absence of a cold environment, BAT is critical for whole-body EE under physiological conditions and long-term fasting (43). To evaluate TRX2 function in BAT under long-term fasting conditions, we fasted mice for 24 hours. We observed histological changes in BAT after long-term fasting similar to those resulting from chronic cold exposure, indicating a requirement for BAT activation in temperature maintenance during fasting (Figure 6A). Additionally, similar lipid metabolic changes, including increased lipid uptake and unresponsive FA oxidation, still existed in BAT of *Trx2^BATKO^* mice after long-term fasting (Figure 6, B and C). However, no significant body temperature change was observed after long-term fasting (Figure 6D).

Next, we measured metabolic parameters of WT and *Trx2^BATKO^* mice during 24 hours of fasting followed by 48 hours of refeeding. *Trx2^BATKO^* mice showed no change in EE or oxygen consumption (VO_2_) compared with WT mice during fasting (Figure 6, E and F). During refeeding, we observed increased EE and VO_2_ and a minor increase in food intake in *Trx2^BATKO^* mice (Figure 6, E–G). Interestingly, despite similar body temperatures, *Trx2^BATKO^* mice showed dramatically increased physical activity relative to WT controls during fasting, but not after refeeding (Figure 6H). However, *Trx2^BATKO^* mice had respiratory exchange ratios (RER) similar to those of WT mice during fasting and refeeding, suggesting similar substrate oxidation under both conditions (Figure 6I).

In conclusion, these findings reflect a compensatory requirement of EE after BAT dysfunction, either from the food during feeding or from skeletal muscle activity during fasting. The contribution of TRX2-deficient BAT to overall EE under physiological conditions is minimal.

### Association of reduced TRX2 expression and decreased FA oxidation in BAT of obese mice.

By analyzing the transcriptome landscape in human adipocytes based on available RNA-Seq data sets from lean, obese, and type 2 diabetes mellitus (T2DM) patients, we have previously revealed reduced TRX2 expression with altered FA metabolism in T2DM adipocytes (29). However, we did not find any available online data on human BAT sequencing in obesity, T2DM, or fatty liver disease due to the scarcity of human BAT. Therefore, we performed RNA microarrays on control versus *ob/ob* mice, a classic animal model with spontaneous obesity, hyperglycemia, and hepatic steatosis. Moreover, *ob/ob* mice exhibit whitening BAT and are cold sensitive (44). A heatmap analysis showed the top 99 significantly upregulated or downregulated genes between cohorts, suggesting an altered transcriptome landscape in obese BAT (Supplemental Figure 3A). Compared with what was seen with the control, the volcano plot displayed differential expression of genes (614 upregulated and 499 downregulated genes) in obese mice (Supplemental Figure 3B). Kyoto Encyclopedia of Genes and Genomes–enriched (KEGG-enriched) pathway analysis indicated a significant downregulation of thermogenesis, FA metabolism, and PPAR signaling in the obese group (https://www.genome.jp/kegg/) (Supplemental Figure 3C). Our microarray data confirmed a downtrend of antioxidant genes (*Trx2*, *Trxr2*, and *Prx3*, *Trx2*, *P* = 0.07) and FA oxidation genes (*Ppara*, *Cpt1b*, *Cpt2*, and *Hadh*; *Ppara*, *P* = 0.017, *Cpt1b*, *P* = 0.03), whereas the expression of proinflammatory cytokine *Tnfa* was markedly elevated (*P* = 0.02). Moreover, BAT from obese mice exhibited reduced expression of TRX2 protein, and mRNA correlated with a decreased PPARα protein and reduced *Cpt1b* mRNA (Supplemental Figure 3, E–G); *Trx2* and *Cpt1b* mRNA levels were negatively associated (Supplemental Figure 3H). Taken together, these data indicated an association of reduced TRX2 expression with decreased FA oxidation in the BAT of obese mice, underscoring the clinical relevance of the *Trx2^BATKO^* mouse model.

### TRX2 loss induces excessive mtROS and cytosolic mtDNA release in BAT.

**Figure 7:**
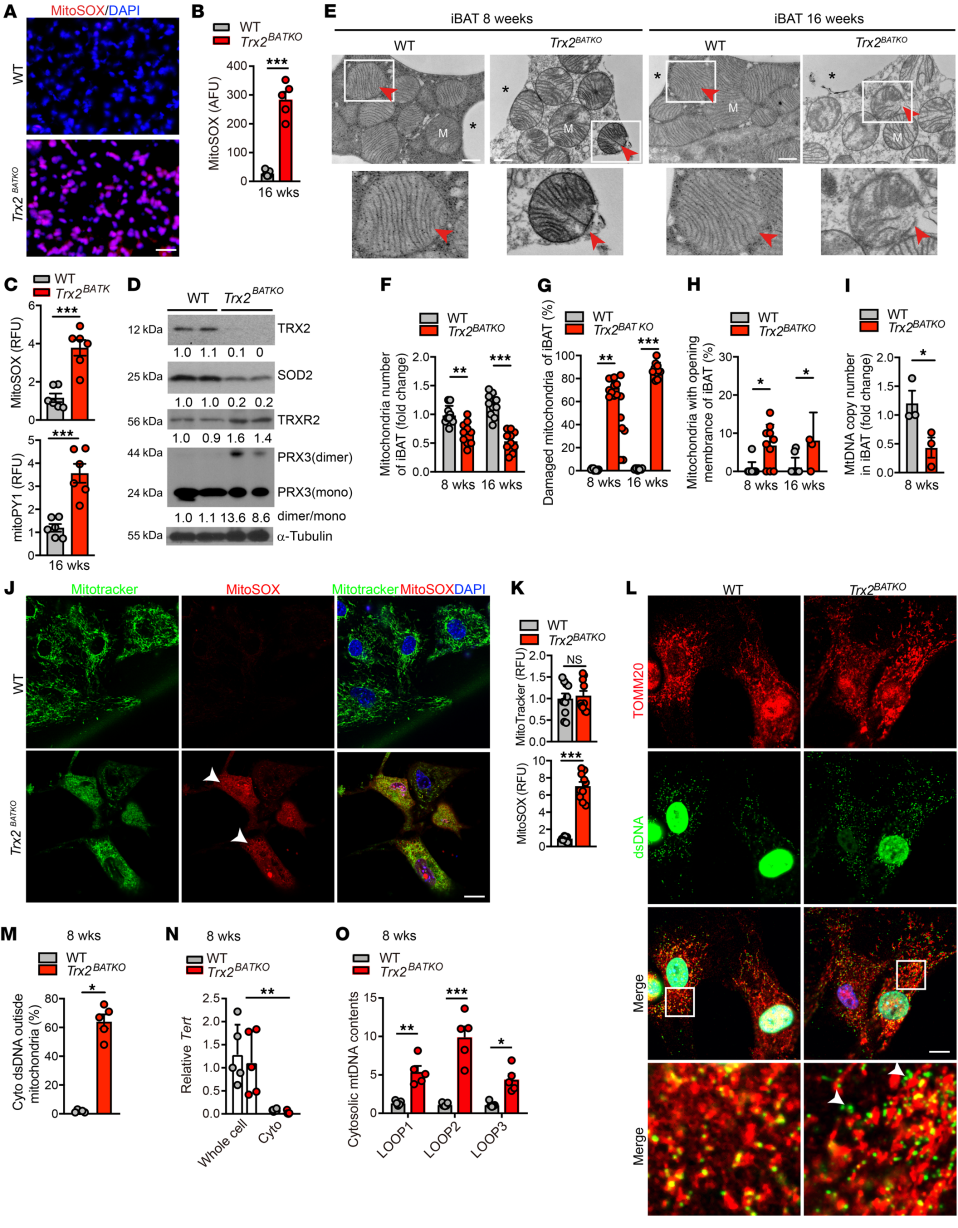
TRX2 loss induces excessive mtROS and cytosolic mtDNA release in BAT. (**A** and **B**) mtROS were detected by MitoSOX in iBAT. Data are presented as arbitrary fluorescence unit (AFU). *n* = 5. (**C**) mtROS were detected by MitoSOX (red) and mitoPY1 (green) in freshly isolated iBAT. Data are presented as relative fluorescence unit (RFU) by taking WT as 1.0. *n* = 6. (**D**) Western blot analysis of redox proteins in BAT. Relative protein levels and PRX3 dimer/monomer ratios are presented as fold changes by taking WT as 1.0. *n* = 2. (**E**–**H**) Mitochondrial structures of iBAT by EM. (**E**) Representative EM images. White boxes denote magnified areas, and arrowheads indicated mitochondria. Quantification of mitochondrial numbers (**F**), damaged mitochondria percentage (**G**), and mitochondria with opening outer membrane (**H**). Ten fields were randomly chosen for each group (*n* = 3). (**I**) mtDNA copy number of iBAT (*n* = 3). *Tert* was used as a nuclei DNA control. (**J** and **K**) mtROS were detected by MitoTracker (green) and MitoSOX (red) in primary brown adipocytes. Arrows indicate MitoSOX*^+^* cells. Quantification of RFU is shown in **K** (*n* = 3). (**L** and **M**) Cytosolic DNA detected by costaining for TOM20 (red) and double-stranded DNA (green) in primary brown adipocytes. Boxes denote magnified areas, and arrows indicate DNA released to the cytoplasm that is quantified in **M**. (**N** and **O**) Cytosolic mtDNA in freshly purified mature brown adipocytes from WT and *Trx2^BATKO^* mice was detected by PCR. (**N**) *Tert* expression. (**O**) Cytosolic mtDNA contents were determined by qPCR with 3 sets of specific primers. Relative mtDNA contents are presented as fold changes by taking WT as 1.0 (*n* = 5). Quantitative data are presented as mean ± SEM. *P < 0.05; **P < 0.01; ***P < 0.001. Significance was assessed by 1-way ANOVA followed by Tukey’s post hoc test (**F**–**H**, **N**, and **O**) or 2-tailed Student’s *t* test (**B**, **C**, **I**, **K**, and **M**). Scale bars: 100 μm (**A**); 0.5 μm (**E**); 10 μm (**J** and **L**). Original magnification for higher magnification images, ×1260 (**E** and **L**).

To determine whether TRX2 deficiency enhances ROS production, we examined mitochondrial oxidative stress levels in situ using MitoSOX, a probe specific for mitochondrial superoxide detection. An excessive amount of mitochondrial superoxide was detected in BAT from *Trx2^BATKO^* mice (Figure 7, A and B). To further measure mtROS in BAT, we detected both superoxide and hydrogen peroxide in fresh BAT tissue with MitoSOX and mitoPY1, respectively. Similarly to superoxide, mitochondrial hydrogen peroxide was increased in *Trx2^BATKO^* BAT compared with WT BAT (Figure 7C). Consistent with the increased mtROS, expression of SOD2, the enzyme-scavenging mitochondrial superoxide, was drastically reduced in BAT from *Trx2^BATKO^* mice. However, PRX3 dimer/monomer ratio, an indicator of oxidative stress and PRX3 functional loss, was highly upregulated in *Trx2^BATKO^* BAT. TRXR2 expression was weakly enhanced, likely due to a compensatory response to increased mtROS in *Trx2^BATKO^* BAT (Figure 7D). Taken together, these data indicate that *Trx2* deficiency in BAT adipocytes induced excessive mtROS, likely by reducing SOD2 expression and increasing PRX3 dimer formation.

We then analyzed the mitochondrial ultrastructure of BAT from WT and *Trx2^BATKO^* mice using transmission electron microscopy. In normal brown adipocytes, mitochondria presented a well-preserved membrane structure with aligned cristae. In stark contrast, in addition to decreased abundance, mitochondria of *Trx2^BATKO^* mice displayed aberrant structure with moderate disorganization of cristae at 8 weeks of age and complete disorganization by 16 weeks. Additionally, we detected disrupted mitochondrial membranes in BAT from *Trx2^BATKO^* mice, shown by a significantly higher percentage of mitochondria with a fractured membrane in an age-dependent manner (Figure 7E with quantifications in Figure 7, F–H). Consistent with the reduced abundance of mitochondria, we found a significant reduction of mtDNA copy number in BAT from *Trx2^BATKO^* mice, indicating that TRX2 deficiency in BAT results in damaged mitochondrial integrity and mass (Figure 7I). Despite these mitochondrial defects, we did not detect any mitophagy in the BAT of *Trx2^BATKO^* mice.

Oxidative stress is a vital factor in cytosolic mtDNA release (45). The obvious disruption of mitochondrial membranes in BAT from *Trx2^BATKO^* mice prompted us to assess mtDNA levels in the cytosol of BAT. Given that adipose tissue contains adipose tissue macrophages, which are an essential contributor to inflammation in WAT (46), we isolated preadipocytes from *Trx2^fl/fl^* (WT) and *Trx2^fl/fl^*;*Ucp1-Cre* mice and differentiated them into brown adipocytes in vitro (Supplemental Figure 4). Similarly to what occurred with BAT, 3- to 4-fold increases of mtROS were detected in the day 8–differentiated Trx2-deficient brown adipocytes compared with WT as measured by MitoSOX (Figure 7, J and K). However, mitochondrial masses as measured by MitoTracker were not significantly different between WT and *Trx2^BATKO^* primary brown adipocytes (Figure 7, J and K). Although in WT, all mtDNAs were colocalized with TOMM20, a mitochondrial marker, the majority of mtDNAs in primary brown adipocytes from *Trx2^BATKO^* mice were outside mitochondria (Figure 7, L and M). We hypothesized that TRX2 deletion enhances mtDNA release to the cytosol. We further purified the cytosolic extracts from the fresh mature brown adipocytes isolated from BAT of WT and *Trx2^BATKO^* mice. Reverse-transcriptase PCR (RT-PCR) confirmed the absence of nucleoli-specific telomerase RNA (*Terc*) in the cytosol (Figure 7N), but a significantly increased mtDNA level in the cytosol of mature brown adipocytes was detected in *Trx2^BATKO^* mice compared with WT, as measured by 3 sets of mtDNA-specific primers (Figure 7O). Taken together, our findings demonstrate that TRX2 deficiency enhances excessive mtROS and triggers the release of mtDNA into the cytosol in BAT.

### TRX2 deletion triggers the cGAS/STING pathway and NLRP3 inflammasome activation in BAT.

**Figure 8:**
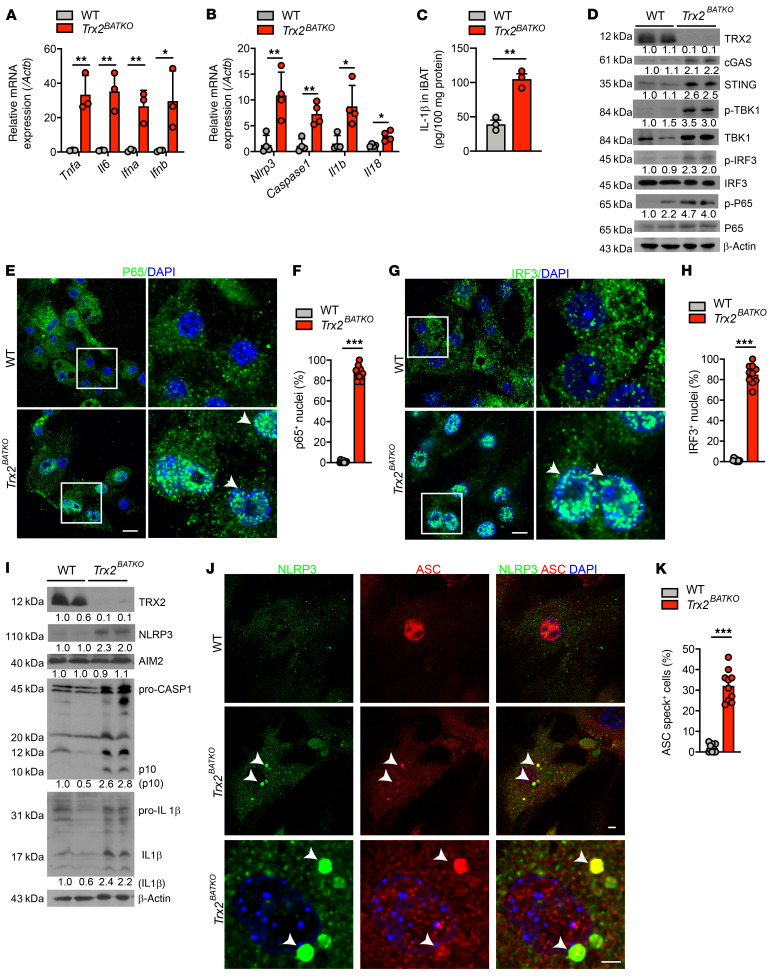
TRX2 deficiency triggers cGAS/STING pathway and NLRP3 inflammasome activation in BAT. (**A**) Proinflammatory cytokine mRNA expression in iBAT from 16-week-old WT and *Trx2^BATKO^* mice (*n* = 3). (**B**) NLRP3 inflammasome-related gene expression of iBAT (*n* = 4). (**C**) IL-1β levels of iBAT as detected by ELISA (*n* = 3). (**D**) Western blots of cGAS/cGAMP/STING pathway proteins in iBAT. Relative protein levels are presented as fold changes by taking WT as 1.0. *n* = 2. (**E** and **F**) Immunostaining of NF-κB p65 (green) in primary brown adipocytes with DAPI counterstaining. Boxes denote magnified areas, and arrowheads denote nuclear p65 or IRF3. Nuclear p65 translocation (% p65*^+^* nuclei) was quantified. (**G** and **H**) Immunostaining of IRF3 (green) in primary brown adipocytes with DAPI counterstaining. Nuclear IRF3 translocation (% IRF3*^+^* nuclei) was quantified. (**I**) Western blots of inflammasome-related proteins in isolated mature brown adipocytes from iBAT. Relative protein levels are presented as fold changes by taking WT as 1.0. *n* = 2. (**J** and **K**) Inflammasome activation in primary brown adipocytes detected by ASC (red) and NLRP3 (green) colocalization. White arrows indicate ASC*^+^* specks. (**K**) Quantification of the percentages of cells with ASC specks in 15 randomly selected fields for each sample. Quantitative data are presented as mean ± SEM. *P < 0.05; **P < 0.01; ***P < 0.001. Significance was assessed by 1-way ANOVA followed by Tukey’s post hoc test (**A**, **C**, **G**, **H**, and **I**). Scale bar: 10 μm (**E**, **G**, and **J**). Original magnification for higher magnification images, ×1260 (**E** and **G**).

Increasing evidence demonstrates cytosolic mtDNA activates inflammation by triggering unchecked innate immune responses, including NF-κB, cGAS/STING, and NLRP3 inflammasome pathways (19, 22, 23). To examine whether the absence of TRX2 induces these pathways, we first determined gene expression of downstream cytokines in BAT of WT and *Trx2^BATKO^* mice. Indeed, we found increased gene expression levels of proinflammatory cytokines, including NF-κB–mediated factors *Tnfa* and *Il6*, as well as cGAS/STING-dependent *Ifnα* and *Ifnβ* in BAT of *Trx2^BATKO^* mice compared with WT (Figure 8A). The NLRP3 inflammasome consists of a sensor (NLRP3), an adaptor (ASC; also known as PYCARD), and an effector (caspase-1). Activation of the NLRP3 inflammasome includes both priming, including transcriptional upregulation of *NLRP3* by signals such as NF-κB, and assembly, in which oligomerized NLRP3 recruits apoptosis-associated speck-like protein containing a caspase-recruitment domain (ASC) to form an ASC speck. Assembled ASC then recruits and activates caspase-1, which in turn cleaves pro–IL-1β and pro–IL-18 (47). Increased mRNA expression of *Nlrp3*, *Caspase1*, *Il1β*, and *Il18* were detected in the BAT of *Trx2^BATKO^* mice (Figure 8B). Additionally, in BAT of *Trx2^BATKO^* mice, mature IL-1β protein was increased (Figure 8C) as was phosphorylation of IFN regulatory factor 3 (IRF3), NF-κB p65, and the upstream TANK-binding kinase 1 (TBK1) (Figure 8D), indicating activation of both the cGAS/STING and the NF-κB pathways. Moreover, p65 and IRF3 staining showed that the cGAS/STING pathway was strongly activated in primary brown adipocytes, as indicated by nuclear translocation of p65 (Figure 8, E and F) and IRF3 (Figure 8, G and H).

Next, we examined inflammasome activation in isolated mature brown adipocytes from BAT of WT and *Trx2^BATKO^* mice. NLRP3 inflammasome activation was evident by increased protein levels of NLRP3, cleaved caspase-1, and mature IL-1β (Figure 8I). Interestingly, the protein expression of absent in melanoma 2 (AIM2), a cytosolic dsDNA sensor known to activate the inflammasome, was not altered after TRX2 deficiency in BAT (Figure 8I). This suggests TRX2 deficiency is responsible for NLRP3-driven inflammasome activation in BAT. *Trx2^BATKO^* primary brown adipocytes also showed augmented NLRP3 activation, as measured by enhanced colocalization of NLRP3 with ASC and formation of ASC specks (Figure 8, J and K). Overall, our findings suggest that TRX2 deletion activates cGAS/STING and NLRP3 inflammasome pathways in BAT.

### TRX2 loss–driven NLRP3 inflammasome activation relies on the mtROS/mtDNA axis in BAT.

To unravel the underlying signaling cascade triggered by TRX2 deficiency, we sought to identify the relationship among mtROS, cytosolic mtDNA, the cGAS/STING pathway, and the NLRP3 inflammasome.

To identify the role of mtROS after TRX2 deletion in BAT, we treated primary brown adipocytes with mitoTEMPO, a pharmacological scavenger of mtROS. As expected, mitoTEMPO protected adipocytes from cytosolic DNA (Supplemental Figure 5, A and B). Moreover, mitoTEMPO blocked activation of the cGAS/STING and the NLRP3 inflammasome pathways, indicated by decreased IRF3 nuclear translocation and reduced ASC-NLRP3 specks (Supplemental Figure 5, B and C). mitoTEMPO also attenuated levels of phospho-p65, STING, cGAS, and NLRP3 in *Trx2^BATKO^* cells (Supplemental Figure 5D). Thus, we conclude that mtROS generated by TRX2 deletion are upstream of mtDNA release, the cGAS/STING axis, and the NLRP3 inflammasome pathways.

**Figure 9:**
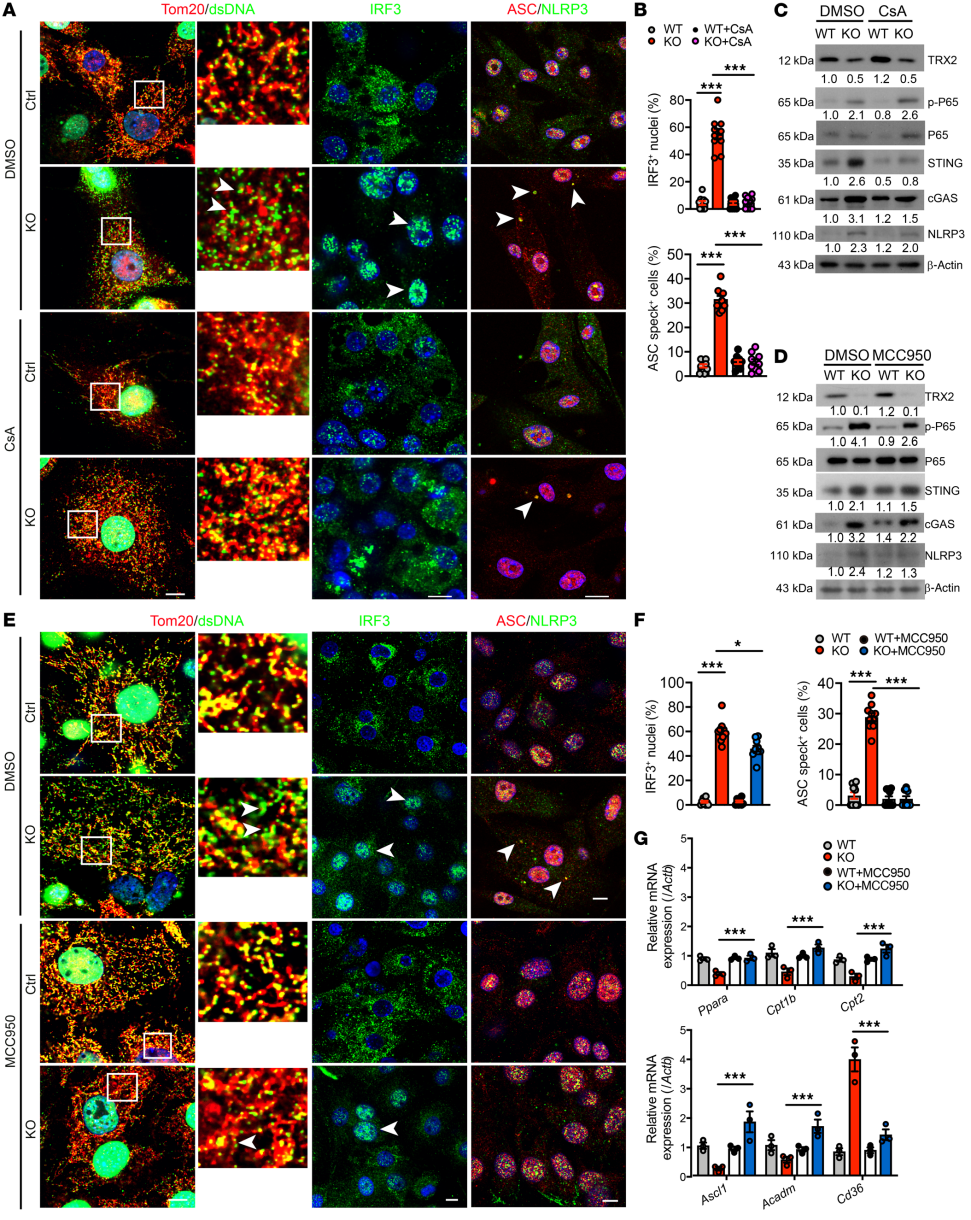
TRX2 loss–driven NLRP3 inflammasome activation relies on mtDNA release in BAT. Primary brown adipocytes were differentiated and cultured in the absence or presence of indicated inhibitors or siRNAs for 4 days. (**A**–**C**) Effects of CsA on mtDNA release and activation of the cGAS/STING and NLRP3 inflammasome pathways. WT and KO primary brown adipocytes were treated with CsA or vehicle (DMSO). (**A**) Immunostaining for cytosolic dsDNA (with mitochondrial marker Tom20), nuclear IRF3, or ASC/NLRP3 specks. Boxes denote magnified areas, and arrows denote cytosolic mtDNAs, nuclear IRF3, and ASC*^+^* specks. (**B**) Nuclear IRF3 translocation (% IRF3*^+^* nuclei) and percentages of ASC speck*^+^* cells were quantified. *n* = 10 random fields. (**C**) Western blots for the cGAS/STING and NLRP3 inflammasome pathways. Relative protein levels are presented as fold changes by taking WT as 1.0. (**D**–**F**) Effects of MCC950 on mtDNA release and activation of the cGAS/STING and NLRP3 inflammasome pathways. WT and KO primary brown adipocytes were treated with MCC950 or vehicle (DMSO). (**D**) Western blots for the cGAS/STING and NLRP3 inflammasome pathways. Relative protein levels are presented as fold changes by taking WT as 1.0. (**E**) Immunostaining of cytosolic dsDNA, IRF3 translocation, and NLRP3-ASC colocalization in primary brown adipocytes treated with MCC950. Boxes denote magnified areas, and arrows denote cytosolic mtDNAs, nuclear IRF3, and ASC*^+^* specks. (**F**) Nuclear IRF3 translocation (% IRF3*^+^* nuclei) and percentages of ASC speck*^+^* cells were quantified. *n* = 10 random fields. (**G**) mRNA expression of lipid metabolism–related genes in WT and KO primary brown adipocytes treated with MCC950 or vehicle. Relative mRNA levels are presented as fold changes by taking WT as 1.0. All experiments were repeated 3 times (*n* = 3). Quantitative data are presented as mean ± SEM. *P < 0.05; **P < 0.01; ***P < 0.001. Significance was assessed by 1-way ANOVA followed by Tukey’s post hoc test. Scale bars: 10 μm. Original magnification for higher magnification images, ×1260.

We further evaluated the role of cytosolic mtDNA release in TRX2 ablation by treating cells with a cyclophilin D inhibitor, cyclosporin A (CsA), which lowers cytosolic levels of mtDNA through inhibition of pore opening (48). Indeed, cytosolic mtDNA release induced by TRX2 deletion was blocked after CsA treatment (Figure 9A). Moreover, decreased IRF3 nuclear translocation (Figure 9, A and B) and reduced expression of cGAS and STING (Figure 9C) indicated the expected cGAS/STING pathway activation was lost after CsA treatment. CsA significantly attenuated NLRP3-ASC specks in TRX2-deficient brown adipocytes (Figure 9, A and B). Reducing cytosolic mtDNA did not diminish, but rather slightly enhanced, phospho-p65 in TRX2-deficient brown adipocytes (Figure 9C). These results support the hypothesis that cytosolic mtDNA release may directly activate NLRP3 inflammasome (49).

As cytosolic mtDNA initiates NLRP3 activation via the cGAS/STING axis (50), we evaluated the cGAS/STING pathway during NLRP3 inflammasome activation. As expected, STING siRNA expression in primary brown adipocytes effectively blocked the cGAS/STING/IRF3 axis and NF-κB (Supplemental Figure 5, E–G), yet had no significant effects on cytosolic mtDNA release or NLRP3 activation (Supplemental Figure 5, F–H). Interestingly, these data suggest that mtDNA escape to the cytosol in brown adipocytes could trigger NLRP3 activation independently of the cGAS/STING and NF-κB pathways. Next, we investigated the NLRP3 inflammasome with the pharmacological NLRP3 inhibitor MCC950. Compared with CsA, MCC950 weakly reduced mtDNA release (Supplemental Figure 5, I and J) and activation of NF-κB and cGAS/STING/IRF3 pathways (Figure 9, D–F). As controls, MCC950 effectively blocked NLRP3 expression and ASC speck formation in primary brown adipocytes (Figure 9, E and F). Collectively, our data showed cytosolic mtDNA release, which further induces the cGAS/STING pathway, is partially dependent on NLRP3 inflammasome activation, indicating a potential positive feedback loop of cytosolic mtDNA and NLRP3. Finally, we determined the effects of inflammasome inhibition on expression of lipid metabolism–related genes. MCC950 rescued the reduced mRNA expression of *Ppara* and its target mitochondrial FA oxidation genes in TRX2-deficient brown adipocytes. Also, MCC950 normalized the increased transcription of *Cd36*, involved in lipid update, in TRX2-deficient brown adipocytes (Figure 9G). Taken together, these data indicate that TRX2 loss in BAT elicited activation of a mtROS/mtDNA-dependent NLRP3 inflammasome pathway, which induced altered lipid metabolism in BAT.

### TRX2 deficiency in BAT promotes ingWAT metabolism without mitochondrial damages or inflammasome activation.

Since ingWAT with multilocular LDs is involved in thermogenesis and can stimulate metabolism by elevating EE (37), we assessed metabolic and inflammatory pathways of ingWAT in *Trx2^BATKO^* mice. Consistent with the intact TRX2 expression, ingWAT of *Trx2^BATKO^* mice had no damaged mitochondria without detectable ROS production or inflammasome activation (Supplemental Figure 6, A–D). *Trx2^BATKO^* mice treated with CL316,243 had normal TRX2 expression, but exhibited augmented induction of ingWAT browning, as evidenced by increased numbers of multilocular adipocytes in ingWAT (Supplemental Figure 6, E–H). These findings indicate that the elevation of multilocular adipocytes in ingWAT is independent of TRX2 expression.

WAT browning, the conversion of subcutaneous white adipocytes to beige adipocytes, is an essential adaptive mechanism in response to cold exposure (37). During cold exposure, FAs are released by WAT and transported into BAT for β oxidation. As expected, *Trx2^BATKO^* mice treated with CL316,243 showed enhanced lipolytic ability of ingWAT and elevated FA oxidation of ingWAT (Supplemental Figure 6, I and J). Moreover, the activity of glucose utilization was augmented in ingWAT of *Trx2^BATKO^* mice, as evidenced by the elevated expression of *Glut4*, *Gck*, and *Pdh1* (see Supplemental Figure 2). Therefore, enhanced utilization of lipid and glucose ingWAT in *Trx2^BATKO^* mice may reflect a compensatory effect of whole-body metabolism in the loss of BAT function. Impaired cold-induced thermogenesis can compensate for WAT lipolysis to mobilize FAs for energy combustion (42, 51). However, in our study, the defective thermogenesis observed after CL316,243 injection suggests that compensatory WAT lipolysis and FA oxidation is insufficient to maintain body temperature in *Trx2^BATKO^* mice. Moreover, our results indicate that lack of TRX2 in BAT activates ingWAT metabolism without mitochondrial damages or inflammasome activation.

### NLRP3 inhibitor MCC950 ameliorates impaired thermogenesis by rescuing defective lipid metabolism.

**Figure 10:**
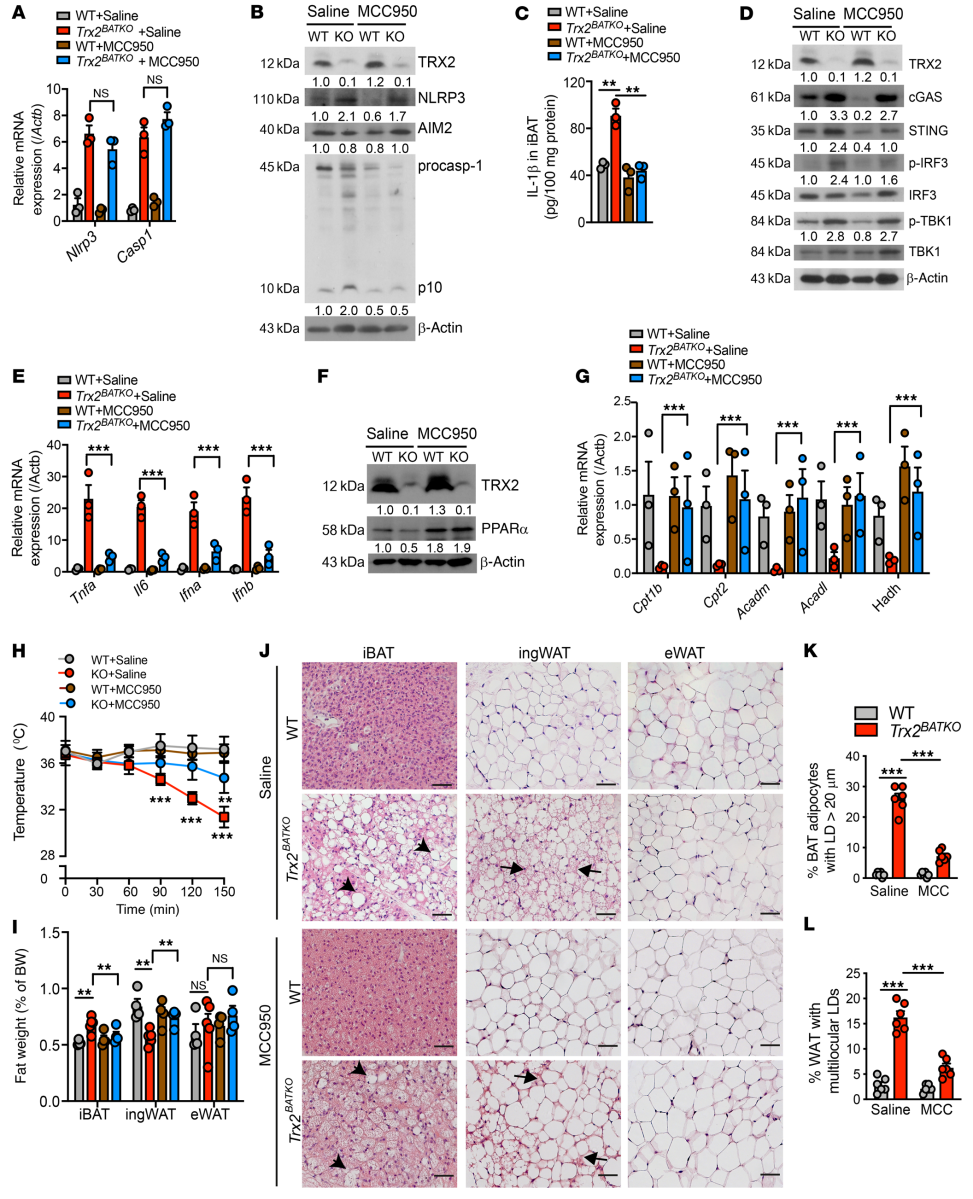
NLRP3 inflammasome inhibitor MCC950 ameliorates the impaired thermogenic phenotype of *Trx2^BATKO^* mice. Eight-week-old WT and *Trx2^BATKO^* mice under NCD were treated with MCC950 (10 mg/kg) or an equal volume of saline by intraperitoneal injection every other day for 8 weeks (*n* = 6). (**A**) mRNA expression of inflammasome-related genes in iBAT (*n* = 3). Relative mRNA levels are presented as fold changes by taking saline-treated WT as 1.0. (**B**) Western blots of NLRP3 inflammasome-related proteins in iBAT. Protein levels are presented as fold changes by taking saline-treated WT as 1.0. *n* = 3. (**C**) Mature IL-1β levels in iBAT were measured by ELISA (*n* = 3). (**D**) Protein levels of the cGAS/STING pathway in iBAT were detected by Western blotting. Protein levels are presented as fold changes by taking saline-treated WT as 1.0. *n* = 3. (**E**) mRNA expression of proinflammatory cytokines in iBAT (*n* = 3). Relative mRNA levels are presented as fold changes by taking saline-treated WT as 1.0. (**F**) Western blots of PPARα in iBAT. *n* = 3. (**G**) mRNA expression of lipid metabolism genes in iBAT (*n* = 3). Normalized mRNA levels (versus β-actin mRNA) are presented. (**H**) Intrarectal temperature during acute cold intolerance test in the absence of food (*n* = 6). (**I**) Ratios of adipose depots to body weight (*n* = 4). (**J**) Representative histologic images of adipose tissues. Arrowheads denote large LDs in BAT, and arrows denote ingWAT with multilocular LDs. (**K** and **L**) BAT adipocytes with unilocular LD diameter of 20 μm or more and percentages of WAT with multilocular LDs are quantified. *n* = 6. Quantitative data are presented as mean ± SEM. *P < 0.05; **P < 0.01; ***P < 0.001. Significance was assessed by 1-way ANOVA followed by Tukey’s post hoc test. Scale bar: 100 μm (**J**).

To determine the role of activated NLRP3 inflammasome in the pathogenesis observed in *Trx2^BATKO^* mice, we treated 8-week-old male mice with the NLRP3 inhibitor MCC950 for 8 weeks. MCC950 efficiently inhibits NLRP3 activation and IL-1β secretion in mice (52, 53). NLRP3 priming was unaffected, as *Nlrp3* and *Caspase1* as well as NLRP3 remained the same in treated and untreated mice (Figure 10, A and B). Importantly, decreased procaspase-1 cleavage and IL-1β protein expression indicated MCC950 efficiently inhibited NLRP3 inflammasome activation (Figure 10, B and C). Consistent with in vitro data, reduced STING and p-IRF3 showed that inhibition of NLRP3 partially suppressed the activated cGAS/STING pathway in *Trx2^BATKO^* mice (Figure 10D). Furthermore, MCC950 partially decreased the expression of proinflammatory cytokines in *Trx2^BATKO^* mice, from approximately 30-fold without treatment to approximately 5-fold after treatment (Figure 10E). Additionally, PPARα protein and FA oxidation gene mRNA expression in BAT of *Trx2^BATKO^* mice recovered to levels similar to those seen in controls after treatment (Figure 10, F and G), indicating an in vivo rescue of altered lipid metabolism. Importantly, MCC950 attenuated the impaired thermogenesis of *Trx2^BATKO^* mice during acute cold exposure (Figure 10H) and in response to CL316,243 (Supplemental Figure 7A). Of note, we did not detect any effects of MCC950 on the CL316,243-induced transient increase in body temperature of either WT or *Trx2^BATKO^* mice. Consistently, the inflammasome activity was only marginally suppressed by CL316,243 at 1 hour in both WT and *Trx2^BATKO^* mice (Supplemental Figure 7B). Taking these data together, we show that CL316,243 induced lipid uptake and lipolysis and its associated increase in the substrate flux, but not inflammasome activity, and contributed to the transient increase in body temperature in CL316,243-induced thermogenesis. Finally, MCC950-treated *Trx2^BATKO^* mice displayed fat mass similar to that of WT mice (Figure 10I). In support, histologic analysis showed that MCC950 reduced the whitening of BAT and browning of ingWAT in *Trx2^BATKO^* mice (Figure 10, J–L), further confirming that the ingWAT browning is a compensatory effect of BAT dysfunction. Overall, these results support a critical role of NLRP3 inflammasome activation in defective lipid metabolism and hypothermia in *Trx2^BATKO^* mice.

### MCC950 treatment reverses the metabolic benefits of Trx2^BATKO^ mice under nutrient overload.

**Figure 11:**
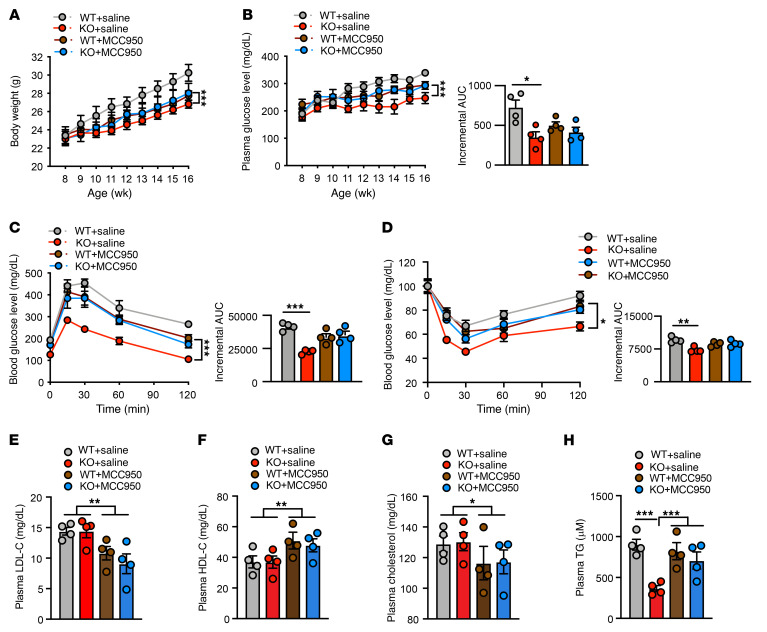
Effects of MCC950 on diet-induced glucose metabolism and insulin sensitivity. Eight-week-old WT and *Trx2^BATKO^* mice were fed with HFD in the presence of MCC950 (10 mg/kg) or an equal volume of saline by intraperitoneal injection every other day for 8 weeks. (**A**) Body weight gain (*n* = 4). (**B**) Fasting blood glucose levels and incremental AUC (*n* = 4). (**C**) Blood glucose levels and incremental AUC in GTT (*n* = 4). (**D**) Blood glucose levels and incremental AUC in insulin tolerance test (*n* = 4). (**E**–**H**) Lipid profiles, including LDL-C (**E**), HDL-C (**F**), TC (**G**), and TG (**H**) (*n* = 4). Significance was assessed by 2-way ANOVA with Bonferroni’s post hoc tests (**A**–**D**) and 1-way ANOVA followed by Tukey’s post hoc test (**E**–**H**).

**Figure 12:**
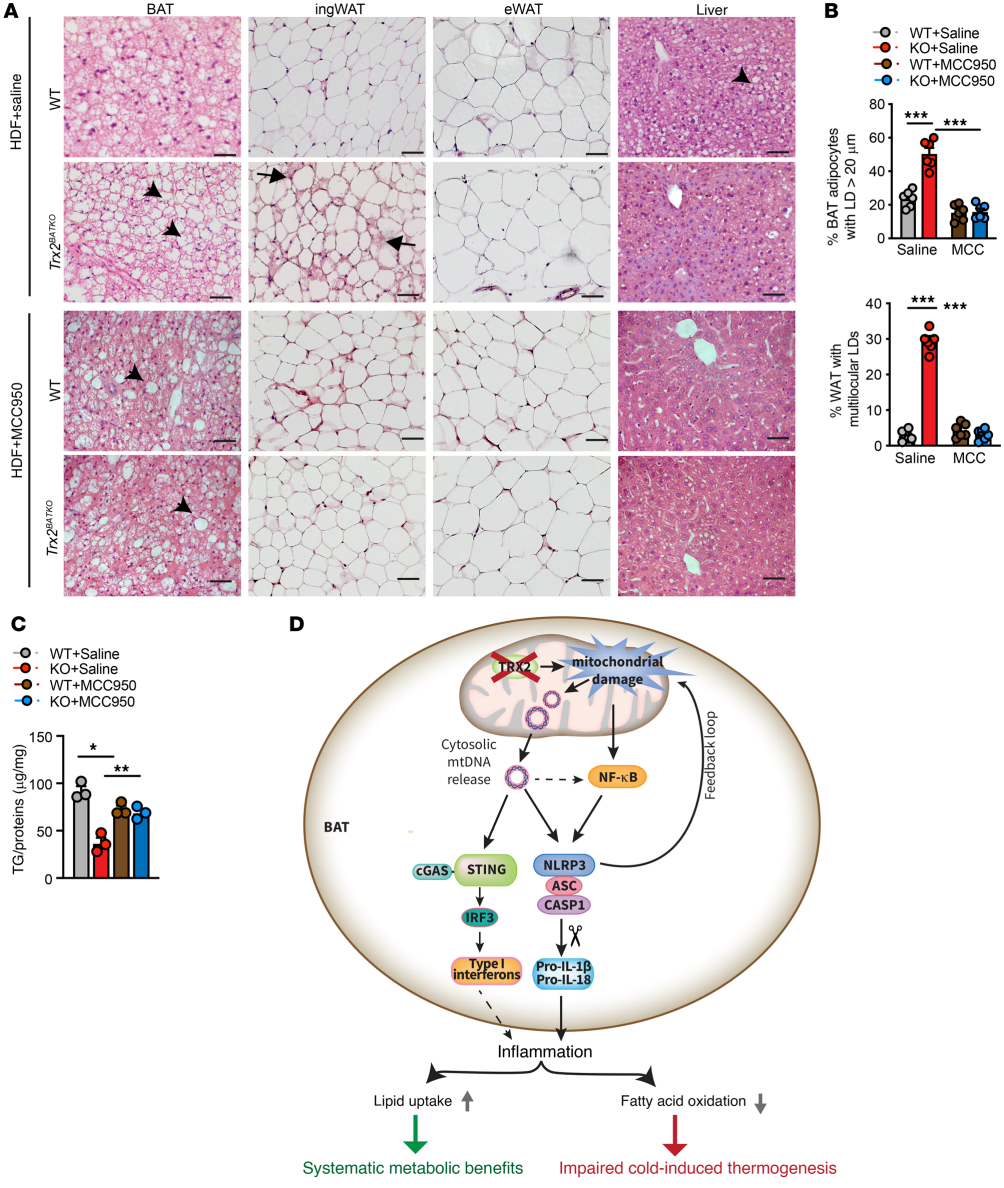
MCC950 treatment reverses the metabolic benefits of *Trx2^BATKO^* mice under nutrient overload. (**A**) Representative histologic images of iBAT, ingWAT, and liver tissues from 16-week-old HFD-fed WT and *Trx2^BATKO^* mice. Arrowheads denote LDs in BAT and liver, whereas arrows denote ingWAT with multilocular LDs. (**B**) BAT adipocytes with unilocular LD diameter of 20 μm or more and proportion of WAT with multilocular LDs are quantified. *n* = 6. (**C**) TG contents of liver from HFD-fed mice with or without MCC950 (*n* = 3). Quantitative data are presented as mean ± SEM. *P < 0.05; **P < 0.01; ***P < 0.001. Significance was assessed by 1-way ANOVA followed by Tukey’s post hoc test (**B** and **C**). Scale bar: 100 μm (**A**). (**D**) A schematic diagram summarizing our findings. TRX2 deficiency in BAT protects from insulin resistance and impairs thermogenesis through the mtDNA/NLRP3 inflammasome pathways. TRX2 ablation in brown adipocytes triggers ROS production and mtDNA release, which induce NLRP3-mediated aberrant innate inflammation in BAT. Consequently, enhanced lipid uptake, with suppressed mitochondrial FA oxidation in BAT, results in the “whitening” of BAT, protecting mice from insulin resistance, but impairing thermogenesis (see text for details).

In *Trx2^BATKO^* mice, activation of the NLRP3 inflammasome in iBAT results in a better metabolic profile under metabolic stress. To explore this further, we administered MCC950 to HFD-fed mice to investigate the impact of brown adipose inflammasome inhibition on whole-body metabolism. MCC950 significantly diminished the ability of *Trx2^BATKO^* to resist diet-induced obesity, as HFD-fed *Trx2^BATKO^* and WT mice had similar body weights after treatment (Figure 11A). HFD-fed *Trx2^BATKO^* mice exhibited lower plasma glucose compared with WT mice under random conditions (Figure 11B) or in the glucose tolerance test (GTT) (Figure 12C), while MCC950 significantly reversed these benefits on glucose metabolism in the HFD-fed *Trx2^BATKO^* mice (Figure 11, B and C). Consistently, MCC950 attenuated the ameliorated insulin sensitivity in the HFD-fed *Trx2^BATKO^* mice (Figure 11D). Furthermore, NLRP3 inhibition ameliorated the hypotriglyceridemia in HFD-fed *Trx2^BATKO^* mice, as seen in the similar plasma TG levels in *Trx2^BATKO^* and WT mice after MCC950 treatment (Figure 11, E–H). Also, histological analyses indicted that MCC950 significantly reversed browning of ingWAT in *Trx2^BATKO^* mice, while eWAT morphology was not altered by Trx2 deficiency or MCC950 (Figure 12, A and B). Although the hepatic steatosis by MCC950 was not evident in H&E staining (Figure 12A), TG content was significantly higher in *Trx2^BATKO^* mice treated with MCC950 compared with the saline group (Figure 12C), suggesting MCC950 reversed the reduced hepatic TG contents in *Trx2^BATKO^* mice. Of note, MCC950 ameliorated HFD-induced hepatic TG accumulation in WT mice, consistent with reports showing that MCC950 improves metabolic and hepatic function in aged mice (54). Therefore, WT and *Trx2^BATKO^* mice treated with MCC950 had comparable hepatic TG contents (Figure 12C). Indeed, MCC950 significantly attenuated the benefits on glucose metabolism in the HFD-fed *Trx2^BATKO^* mice and improved the glucose tolerance of HFD-fed WT mice (see Figure 11, B–D). Nevertheless, our data support an important role of NLRP3 inflammasome activity in balancing lipid metabolism, as MCC950 administration to HFD-fed *Trx2^BATKO^* mice diminished TRX2 deletion-induced metabolic benefits.

## Discussion

Despite the mounting evidence of metabolic stress–induced BAT inflammation and its effect on disrupting thermogenesis (2–4), the contribution of BAT inflammation to systematic metabolism has not been established. Here, we report on a transgenic mouse model for examining the effects of BAT-specific deficiency of TRX2, a mitochondrial redox protein that scavenges mtROS. We found that TRX2 deficiency in brown adipocytes impaired thermogenesis while benefiting whole-body metabolism by protecting from diet-induced adiposity, hypertriglyceridemia, and hepatic steatosis. Loss of TRX2 disrupted mitochondrial integrity and function, suppressed mitochondrial FA oxidation, and enhanced lipid uptake, resulting in the “whitening” of BAT. More importantly, the effects of TRX2 deletion are caused by unchecked amounts of mtROS, which trigger release of mtDNA into the cytosol. These 2 parallel pathways activated the NLRP3 inflammasome. The excess mtROS activated NF-κB signaling, which contributed to priming of the NLRP3 inflammasome, while cytosolic mtDNAs directly induced the NLRP3 inflammasome in a cGAS/STING-independent manner. We found that NLRP3 inflammasome-mediated production of cytokines IL-1β and IL-18 exacerbated the development of chronic sterile inflammation in BAT of *Trx2^BATKO^* mice. In addition, in vivo pharmacological inhibition of NLRP3 using MCC950 effectively dampened NLRP3 inflammasome activation, recovered impaired lipid metabolism, and improved the hypothermia phenotype in *Trx2^BATKO^* mice. Our data not only identify an adaptive mechanism of BAT under metabolic stress, but also highlight theTRX2-mtDNA-NLRP3 inflammasome as a crucial hub integrating oxidative stress, inflammation, and whole-body metabolic homeostasis (Figure 12D). *Trx2^ADKO^* mice develop a type 2 diabetic phenotype with hepatic insulin resistance, and a HFD exacerbates hepatic steatosis with increased hepatic lipogenesis and gluconeogenesis observed in these mice (29). Excessive ROS induces mitophagy in WAT, but not BAT, of *Trx2^ADKO^* or *Trx2^BATKO^* mice. The exact mechanism for the distinct outcomes in different cell types from TRX2 deletion and excessive ROS remains to be determined. We postulate that this may be related to mitochondrial abundance and/or the ratio of mitophagy machinery to mitochondria in each cell type.

Recent independent studies have linked BAT defects with an adaptive response to metabolic stress, distinct from its role in adaptive thermogenesis, which counteracts obesity and insulin resistance (8, 9). A possible mechanism is the reduction of mtDNA-encoded ETC gene expression, which could lead to defective adaptive thermogenesis, but reduced adiposity and hepatic steatosis at RT or thermoneutrality (9). Consistent with these data, our study shows that *Trx2^BATKO^* mice, while cold intolerant, are resistant to diet-induced obesity, insulin resistance, and hypertriglyceridemia. Of the altered lipid metabolic pathways after TRX2 deficiency, defective FA oxidation appears to be the primary cause of impaired thermogenesis, evident by the unresponsiveness of FA oxidation after cold exposure. In line with our data, CPT2^A−/−^ mice, whose BAT lacks FA oxidation, show impaired adaptive thermogenesis (55). However, the complicated phenotype of *Trx2^BATKO^* mice could not be explained by impaired FA oxidation alone, since CPT2^A−/−^ mice did not show metabolic benefits (55). Alternatively, given the powerful capacity of BAT in clear circulating TGs, increased lipid uptake in BAT might contribute to the metabolic improvement of *Trx2^BATKO^* mice. A number of studies have supported this theory including the following: (a) adipose Lpl–deficient mice showed elevated plasma TG levels associated with reduced lipid uptake into BAT (56); (b) BAT Angptl4-deficient mice performed better in GTT and insulin sensitivity tests (57). Together, these data suggest that dysregulation of more than one lipid metabolism pathway may be involved in connecting BAT dysfunction to the systematic metabolic benefits observed in *Trx2^BATKO^* mice. Yet the upstream mechanism regulating lipid metabolism remains unexplored. Our study reveals local BAT inflammation as an upstream mechanism, activated in response to metabolic stress, which regulates local lipid metabolism and further benefits systemic metabolism. On the other hand, metabolic benefits of *Trx2^BATKO^* mice might be related to the metabolic compensation of other tissues, evidenced by the increased glucose and lipid metabolism of liver, muscle, and WAT. Similarly to our data, it has previously been shown that CGI-58 BAT–deficient mice, which have defective lipolysis in BAT, show improved glucose tolerance along with enhanced browning of ingWAT (51). One possible explanation is that BAT inability to utilize nutrients drives the substrate flux flowing dynamically to other tissues and, being utilized, resulting in a better metabolic performance. More importantly, we found a marked reduction of TRX2 expression in BAT from *ob/ob* mice, suggesting a potential clinical association between TRX2 in BAT and metabolic diseases.

Finally, the connection among BAT inflammation balance, impaired adaptive thermogenesis, and systematic metabolic benefits remains unknown. One possible connection between BAT inflammation and altered lipid metabolism is the inactivation of PPARα. PPARα plays pivotal roles in the regulation of lipid metabolism. PPARα activation in BAT not only directly upregulates FA oxidation genes to induce thermogenesis, but also regulates other lipid metabolic pathways, including lipogenesis and lipoprotein uptake. PPARα directly targets *Angptl4* in hepatocytes and adipocytes, and mice lacking *Angptl4* in BAT show enhanced lipid uptake and improved glucose tolerance (57). Moreover, decreased *Angptl4* translation impairs lipolysis in adipose tissues (58). In our study of TRX2 deficiency in BAT, several pieces of evidence suggested that PPARα inactivation may link inflammation and lipid metabolism. First, TRX2 ablation downregulated PPARα expression, its target mitochondrial FA β-oxidation genes, and *Angptl4*. Second, pharmacological inhibitors of the mtROS/mtDNA/NLRP3 axis in primary brown adipocytes upregulated the suppressed expression of PPARα. Finally, in vivo inhibition of the NLRP3 inflammasome rescued both the hypothermia phenotype during cold exposure and expression of PPARα and its downstream lipid metabolic genes, which is consistent with recent data on MCC950 activation of PPARα expression in aged mice (54). How the inflammasome regulates PPARα remains unclear and needs more investigation.

In summary, our data demonstrate a strong relationship between the innate immune response in BAT and systematic metabolism. Inflammation in BAT may be an adaptive response to metabolic stress, providing protection from obesity-induced insulin resistance while also impairing thermogenesis. Investigating innate immunity’s role in regulating whole-body metabolism will not only add a further dimension to our understanding of BAT physiology, but will also provide novel therapeutic insight into metabolic disorders.

## Methods

All materials and methods have been either published previously or are presented in the Supplemental Methods, including details regarding animal models, GTT, insulin tolerance test, evaluation of metabolic phenotype, intestine lipid absorption, in vivo and ex vivo lipolysis assays, adipose tissue FA oxidation, thermogenesis assays, and assessment of mitochondrial dynamics. Real-time PCR primers, antibodies, and cytokines used in this study are listed in Supplemental Table 1, 2, and 3, respectively.

### Whole-genome microarray.

Microarray was performed to compare differentially expressed genes (DEGs) in BAT between control and obese mice. Microarray profiling data have been uploaded to the NCBI’s Gene Expression Omnibus database (GEO GSE191009).

### Study approval.

All animal procedures were approved by the Yale University Animal Care and Use Committee.

### Statistics.

All quantitative data are expressed as mean ± SEM. Two-group comparisons were carried out using unpaired 2-tailed Student’s *t* test. Multigroup comparisons were carried out using 1-way ANOVA with Tukey’s post hoc tests. When both genotypes and treatments were considered, comparisons were carried out using 2-way ANOVA with Bonferroni’s post hoc tests. *P* < 0.05 was considered statistically significant.

## Author contributions

YH, JHZ, and HZ performed in vivo and in vitro experiments. RJP and GIS performed experiments for EE. ACD, AKS and CFH performed experiments for lipid uptake and β-oxidation. WM and CFH supervised the entire project.

## Supplementary Material

Supplemental data

## Version 1 02/24/2022

In-Press Preview

## Version 2 05/02/2022

Electronic publication
