# Supplementary material for: Brown adipose TRX2 deficiency activates mtDNA-NLRP3 to impair thermogenesis and protect against diet-induced insulin resistance
Source: J Clin Invest. 2022 May 2;132(9):e148852. doi: 10.1172/JCI148852 (PMC9057632; doi:10.1172/JCI148852)
Supplement: Supplemental data [file jci-132-148852-s154.pdf]

SUPPLEMENTAL DATA

Brown adipose TRX2 deficiency activates mtDNA-NLRP3 to impair thermogenesis and protect against diet-induced insulin resistance

Yanrui Huang[1], Jenny Huanjiao Zhou[1], Haifeng Zhang[1], Alberto Canfran-duque[2], Abhishek Singh[2], Rachel J. Perry[3], Gerald I. Shulman[3], Carlos Fernandez-Hernando[1,2], Wang Min[1]

[1]Interdepartmental Program in Vascular Biology and Therapeutics, Department of Pathology, Yale University School of Medicine, New Haven, 06520, Connecticut, USA.

[2]Interdepartmental Program in Vascular Biology and Therapeutics, Department of Comparative Medicine, Yale University School of Medicine, New Haven, 06520, Connecticut, USA.

[3] Department of Internal Medicine, Yale School of Medicine, New Haven, 06520, Connecticut, USA.

SUPPLEMENTAL MATERIALS AND METHODS

Animal models. Brown adipocyte-specific TRX2 knockout mice (*Trx2^{BATKO}*) were generated by crossing *Trx2$^{lox/lox}$* mice produced in our lab previously (1) with *Ucp1-cre* mice (The Jackson Laboratory, B6.FVB-Tg (Ucp1-cre)1Evdr/J; Stock# 024670). All mice had been backcrossed with C57BL/6 mice for more than 10 generations. The knockout of *Trx2* in adipocytes was verified by real-time PCR (RT-PCR) with primers amplifying *Trx2* exon 3 (1). *Trx2*$^{lox/lox}$ littermates without Ucp1-cre transgene were used as wild-type controls (WT) for all the experiments. Mice were provided with free access to food and water and maintained in a pathogen-free animal facility at 22℃ with a 12 h/12 h light/dark cycle from 7 AM to 7 PM. Mice were fed with normal chow diet (NCD) consist of consist of 10% kcal fat, 70% kcal carbohydrates, and 20% kcal protein (D12450B, Research Diets, Inc). For the high-fat diet experiment, 8-week-old mice were fed with high-fat diet (HFD; D12451, Research Diets, Inc) containing 45% energy from fat, 35% energy from carbohydrates, and 20% energy from protein for 8 weeks. Male and female animals were used in equal numbers for baseline experiments, and both male and female exhibited similar phenotypes in BAT. We used only males for further experiments and littermates were compared in all studies. Diet or drug treatments were started at 8 weeks of age. For tissue collection, we sacrificed mice and perfused them with ice-cold PBS. Different adipose tissue depots (inguinal, epididymal, retroperitoneal WAT and interscapular, subscapular, cervical BAT), heart, liver, kidney, pancreas, and skeletal muscles were collected, weighed, and immediately frozen in liquid nitrogen.

Body composition and metabolic analysis. Metabolic cage evaluation was performed by the Yale Mouse Metabolic Phenotyping Center. After a 2-day acclimation period, mice were fasted for 24 h, followed by refeeding for 48 hours. Indirect Calorimetry (Oxymax/ CLAMS, Columbus Instruments, Columbus, OH) was used to measure energy expenditure, oxygen consumption (VO2), carbon dioxide production (VCO2), food intake, and physical activity of mice. The respiratory exchange ratio (RER) was calculated as VCO2/VO2. Fat mass, lean mass, and percentage of fat were determined by using EchoMRI Body Composition Analyzer (EchoMRI LLC.).

Glucose tolerance test and insulin tolerance test. For the glucose tolerance test (GTT), mice were fasted overnight (16 hours). Basal glucose level was evaluated, and mice were intraperitoneally injected with dextrose (1 g/kg body weight). For the insulin tolerance test, after a 6-hour fast, mice were administered insulin (0.35 U/kg body weight for NCD-fed mice, 0.6 U/kg body weight for HFD-fed mice) by intraperitoneal injection. Blood glucose levels were determined 15, 30, 60, and 120 minutes after injection from tail veins with a glucometer (LifeScan).

Histology and immunofluorescence. Adipose and liver tissues were formalin-fixed, paraffin-embedded, and cut into 5-μm thick sections. Tissue sections were stained with hematoxylin and eosin and viewed on a light microscope. White adipocyte size was analyzed using NIH ImageJ software. Immunofluorescence staining was conducted according to standard protocols. TUNEL (Roche) staining was performed following the manufacturer's instructions. Bodipy (Invitrogen) staining was performed on 10-μm thick liver cryostat sections. Sections were rinsed with PBS and stained with 1 μg/mL BODIPY at room temperature for 15 min. Fluorescent images were acquired under a fluorescence microscope.

Evaluation of metabolic phenotype. Plasma was isolated from blood samples drawn from overnight-fasted (16 hours) mice under anesthesia. Lipid profiles, including the HDL-C, LDL-C, total cholesterol, and tissue triglyceride contents, were evaluated by the Yale Mouse Metabolic Phenotyping Center with a Cobas Mira analyzer (Roche Diagnostics). Enzyme-linked

immunosorbent assay (ELISA) kits were used to determine levels of plasma insulin (ALPCO) and plasma/tissue IL-1β (R&D Systems). Colorimetric/fluorometric kits were used to determine plasma levels of triglyceride (Abcam) and non-esterified fatty acids (BioVision).

***In vitro* glycolysis.** The glycolysis by differentiating brown adipocytes was measured using a Glycolysis Assay Kit (Abnova, China) according to the manufacturer's instructions. In this assay, L-Lactate, the main product of glycolysis, is metabolized to NADH, which reduces formazan dye. Therefore, the signal of reduced dye is proportional to glycolysis by cells. 3T3-L1 adipocytes were transfected with *Trx2* sgRNA or negative control. By Day-4 of differentiation, culture mediums were isolated to measure the intensity of the reduced dye by reading the OD ratio at wavelength 565 nm.

Oral fat tolerance test. The fat tolerance test was performed as previously described (2). After being fasted for 4 hours beginning at 7 AM, mice were administered with oral gavage of olive oil (10 µl/g body weight). Blood samples were drawn from the tail vein 0, 1, 2, and 4 hours after administration. Plasma triglyceride levels at each time point were determined using a commercial assay Kit (Wake).

Tissue lipid uptake. Quantification of lipid uptake in tissues was performed previously described (3). The following emulsion was prepared 1 hour before administration: two µCi [H^3]-triolein and 100 µl of mouse intralipid 20% emulsion oil sonicated on ice for 10 min at 100 W, 0.5-pulse mode to generate micelles. Mice were fasted for 4 hours, starting at 7 AM, followed by oral gavage of the emulsion above. Two hours after administration, plasma/tissues were collected from the euthanized mice. Similar amounts of tissues (100-200mg) were weighted, and lipids were extracted using isopropyl alcohol/hexane mix (2:3 v/v). The lipid layer was then moved to a new glass tube and evaporated. The radioactivity of [^{3}H]-cholesterol was analyzed by liquid scintillation counting. Plasma radioactivity was used as a reverse indicator for total tissue lipid uptake.

Intestine lipid absorption. Mice were fasted at 7AM for 4 hours before being injected with LPL inhibitor poloxamer 407 (1g/kg body weight) to inhibit triglyceride-rich lipoprotein (TRL) catabolism. Two hours after injection, mice were administered the [H^3]-triolein emulsion above. Blood samples were collected 1, 2, and 4 hours after administration. The radioactivity of [^{3}H]-cholesterol in the plasma was analyzed.

Hepatic VLDL-TAG secretion. Hepatic VLDL-TAG secretion was measured as previously described (2). Briefly, mice were fasted after overnight before intraperitoneal injection with poloxamer 407 (1g/kg body weight) to suppress lipolysis of TRL. Blood samples were collected 0, 1, 2, and 4 hours after injection. Plasma triglyceride concentrations were determined using the triglyceride assay kit (Wake).

Cold exposure test. For the acute cold exposure test, mice were fasted for 4 hours before the experiment, starting at 7 AM. Mice were then kept in a cold room at 4°C without food, and rectal temperature was monitored using an ATC1000 Animal Temperature Controller (World Precision Instruments) every 30 minutes. For the chronic cold exposure test, mice were kept in a cold room at 4°C, starting at 11 AM with free access to food and water for 72 hours.

***In vivo* lipolysis.** In vivo lipolysis assays were performed as previously described (2). Mice were fasted for 7 hours starting at 7 AM, and then injected intraperitoneally with saline. After 15 minutes, blood samples were collected through the tail vein. After 2 days, 7 hours fasted mice were intraperitoneally injected with isoproterenol (10 mg/kg body weight), and blood was collected 15

minutes after injection. Plasma concentrations of NEFA were analyzed using a NEFA-C-kit (Wako).

***Ex vivo* lipolysis.** Freshly isolated adipose depots (~20 mg) were incubated in 500 μL DMEM containing 2% fatty acid-free BSA with or without 10 μM isoproterenol for 60 minutes at 37°C. After incubation, the medium was collected for the detection of FFA using a NEFA-C-kit (Wako). FFA release ratio were normalized to FFA levels without ISO.

Adipose tissue FA oxidation. FA oxidation was determined as previously described (2). In brief, freshly isolated adipose tissues were homogenized in ice-cold STE buffer (pH 7.4, 0.25 M sucrose, 10 mM Tris-HCl, and 1 mM EDTA) and then centrifuged. The pellets were resuspended and placed in an incubation chamber with the reaction mixture containing 0.5 mmol/L palmitate (supplemented with 0.4 μCi/mL of [14C] palmitate and 7% BSA) for 30 minutes. The mixture was then transferred to a new tube with filter paper presoaked with 1 mol/L sodium hydroxide. The tubes were incubated at 37°C for 1 hour. Trapped 14-carbon dioxide was then released and collected onto the filter paper. Radioactivity was quantified by liquid scintillation counting.

CL-316,243 treatment. Chronic CL-316,243 injection was performed as previously described (4). Mice were intraperitoneally injected with CL-316,243 (1 mg/kg body weight) or saline daily at 10 AM for 4 days and sacrificed around 1 PM on day 5. On the first day after CL injection, serum NEFA, serum glycerol, and intrarectal temperature was measured.

Transmission electron microscopy. To prepare tissue for electron microscope (EM), we isolated adipose tissues from the mice and immediately cut them into small pieces (1 μm × 1 μm × 1 μm) in ice-cold PBS. Samples were gently shaken with EM fixing buffer (2.5% glutaraldehyde and 2% paraformaldehyde [pH 7.4] in 0.1M sodium cacodylate) at room temperature for 30 minutes and then incubated at 4° C for 90 minutes, followed by treatment with EM wash buffer (0.1 M sodium cacodylate buffer [pH 7.4]). Subsequent processing was carried out by the Yale EM facility. Samples were viewed in an FEI Tecnai BioTWIN transmission EM with an accelerating voltage of 80 kV, and images were obtained using an SIS Morada 11-megapixel charge-coupled device camera and iTEM software (Olympus).

Real-time-PCR analysis and mitochondrial DNA copy number quantification. Total RNA was extracted from tissues and primary adipocytes using TRIzol (Invitrogen). Complementary DNA was synthesized from total RNA by the iScript cDNA Synthesis Kit (Bio-Rad). Real-time-PCR was performed on an iCycler Real-Time Detection System (Bio-Rad) with iQ SYBR Green Supermix (Bio-Rad) and gene-specific primers. Target mRNA gene expression was normalized to *Actb*. Total DNA was extracted from tissues and primary adipocytes using DNeasy Blood & Tissue Kit (QIAGEN). Target mitochondrial DNA copy number was normalized to nuclear gene *Tert*. Real-time-PCR primers are listed in **Table S1**.

Western blot analysis. Western blot analysis was performed as previously described (1). Tissues were homogenized using an ultrasonic homogenizer. To avoid fat contamination, we used 25G1/2 syringes to collect the subnatant of the homogenate after centrifugation. For OXPHOS expression analysis, protein samples were warmed at 37°C instead of boiled at 100°C to avoid degradation of the heat-sensitive complexes. The antibodies used are listed in the **Table S2**.

Assessment of mitochondrial dynamics. mtDNA copy number was quantified as previously described (1). Briefly, total DNA was extracted using Dneasy Blood & Tissue Kit (QIAGEN). Quantitative PCR was performed using nuclear DNA primers (*Tert*) and mtDNA primer (mtDNA

loop1). The *Tert* DNA values served as normalization controls for the mtDNA values. To determine ATP generation, we isolated mitochondria from freshly collected BAT using the Mitochondria Isolation Kit for Tissue (Thermo Scientific) according to the manufacturer's instructions. The isolated mitochondria were then assessed with an ATP bioluminescent assay kit (Sigma) following the manufacturer's protocol.

Mitochondrial ROS production and mitochondrial membrane potential. Detection of tissue mtROS production was performed as previously described (1). Briefly, freshly collected BAT was immediately cut into 30-μm cryostat sections without fixation. The fresh sections were then stained with 2.5 μM MitoSOX (Invitrogen) at 37°C for 10 minutes and visualized immediately using a fluorescent microscope (Zeiss). Quantification of fluorescence intensity was measured by examining six random fields per section. To detect the mitochondrial ROS production of primary brown adipocytes, we incubated cells with MEM supplemented with 2.5 μM MitoSOX, 500 nM MitoTracker Green (Invitrogen) and 1.76 μM Hoechst dye 33342 (Invitrogen) at 37°C for 10 minutes. Images were obtained using a confocal microscope (Leica SP5).

Detection of mtDNA in cytosolic DNA extracts. Cytosolic DNA extraction was performed as previously described (5). Freshly purified mouse mature adipocytes were divided in two aliquots of equal volume. One aliquot was used for extracting total DNA by Dneasy Blood & Tissue Kit (QIAGEN). One aliquot was used for extracting cytosolic DNA using the digitonin methods. Briefly, the adipocytes were resuspended in 500 μL cytosolic DNA extraction buffer (150 mM NaCl, 50 mM HEPES (pH 7.4), and 25 μg/mL digitonin) and gently shaken in room temperature for 10 minutes to allow for selective cytoplasmic membrane permeabilization. The homogenates were centrifuged three times at 1,000x g for 5 minutes. The first pellet was used as the "P" fraction for confirmation immunoblots. The supernatant was transferred to a new tube and centrifuged at 17,000x g for 25 minutes to eliminate contamination from any remaining cellular debris, including nuclear, mitochondrial, and endoplasmic reticulum. Cytosolic DNA was then isolated from these cytosolic extracts using Dneasy Blood & Tissue Kit. Quantitative PCR was performed on both total DNA extracts and cytosolic DNA extracts using nuclear DNA primers (*Tert*) and mtDNA primers (mtDNA loop1 to 3). The mtDNA values obtained from total DNA extracts were used as normalization controls for the mtDNA absorbance obtained from the cytosolic DNA extracts. The *Tert* DNA values in cytosolic DNA extracts served as detectors for nuclear contamination in the cytosolic DNA extracts.

Primary brown adipocytes isolation, purification, and differentiation. Freshly isolated iBAT was minced and digested with 1 mg/mL collagenase type 2 (Hank's buffered salt solution supplemented with 4% FA-free BSA and 2.5% penicillin/streptomycin) in a 37°C water bath for 50 minutes. After digestion, the mixture was filtered through a 100-μm filter to remove undigested tissue, and then centrifuged for 10 minutes at 1200 rpm. The floating cell layer on the top of the tube was carefully collected as mature brown adipocytes.

The cell pellet containing preadipocytes was then resuspended in growth medium (minimum essential medium, 10% FBS, 1% penicillin/streptomycin, 25 mM $NaHCO_3$) and plated onto a 10-cm cell culture dish. The medium was refreshed every 3 days. After reaching confluence, the cells were trypsinized and seeded onto 6-well cell culture plates in growth medium. The differentiation of primary brown adipocytes was performed as previously described (6). Briefly, after being held at confluence for 2-3 days without changing the medium, the cells (day 0) were cultured in adipogenic I medium (growth medium supplemented with 0.5mM 8-Methoxymethyl-3-isobutyl-1-methylxanthine [IBMX], 1.7 μM insulin, and 1 μM dexamethasone) for 2 days. The cells were then switched to adipogenic II medium (growth medium supplemented with 17 nM Insulin, 2 μM Troglitazone, 1 μM Rosiglitazone and 1 nM 3,3′,5-triiodo-L-thyronine) for 6-8 days until the cells were fully differentiated. At different time points during differentiation (day 0, 2, 4, or 8), *Ucp1*-cre

was expressed in mature brown adipocytes from *Trx2*$^{lox/lox}$; *Ucp1*-cre mice, specifically deleting the *Trx2* gene in these cells but not in mature brown adipocytes from WT (*Trx2*$^{lox/lox}$) mice. Cells were subjected to various assays.

Seahorse oxygen consumption assay in primary brown adipocytes. Macrophage-negative SVF isolated from iBAT were seeded into each well of gelatin-coated XF96 cell culture microplates and differentiated into mature brown adipocytes. On the day of experiment, cells were rinsed twice and then cultured in Agilent Seahorse XF Base Medium with 25 mM glucose and 1 mM pyruvate in a non-CO2 incubator at 37°C for 1 hour. The microplates were then transferred to a Seahorse XF96 analyzer to perform the mitochondrial stress test. After three basal respiration measurements of the cells were obtained, the coupled respiration inhibitor oligomycin (1 μM) was added to assess ATP production and proton leak. The maximal respiration of the cells was determined after adding carbonyl cyanide-4-(trifluoromethoxy) phenylhydrazone (FCCP) (600 nM). Lastly, non-mitochondrial respiration was measured by adding 0.5 μM rotenone and 0.5 μM antimycin A. Results were analyzed using the Wave software (Agilent Technologies).

Immunofluorescent staining. Cultured primary brown adipocytes cells were fixed with paraformaldehyde, permeabilized with 0.1% Triton X-100, blocked, and incubated at 4°C with the primary antibodies overnight. After incubation with corresponding secondary antibodies, cells were viewed on a confocal microscope (Leica SP5). In the dsDNA immunofluorescent staining, cells were permeabilized with digitonin, a selective plasma permeabilization buffer, to prevent contamination of mitochondrial and nuclear DNA in the cytoplasm. TOMM20, a protein located in the mitochondrial outer membrane, was co-stained with dsDNA and used as a mitochondrial marker.

Inhibition of mtROS, mtDNA release, STING, and NLRP3. On the day of differentiation, primary brown adipocytes were treated with the mitochondria-specific antioxidant mitoTEMPO (25 μM), cyclosporin A (10 μM), or NLRP3-specific inhibitor MCC950 (10 μM) in adipogenic I medium. On day 2, the culture medium was changed to adipogenic II medium containing mitoTEMPO or cyclosporin A or MCC950. On day 4, the cells were harvested for immunofluorescent staining, cytosolic mtDNA detection, and western blot analysis.

For STING pathway inhibition, primary brown adipocytes were transfected with STING siRNA (40 nM) (Santa Cruz) on day 2 during differentiation. Control primary brown adipocytes were transfected with scrambled siRNA. After 48 hours, cells were harvested for further analysis.

For NLRP3 activation inhibition *in vivo*, 8-week-old WT and *Trx2*^{BATKO} mice under a NCD or HFD were treated with MCC950 (10mg/kg) or an equal volume of saline by intraperitoneal injection every other day for 8 weeks (n=6 per group). Body weights and random plasma glucose levels were measured weekly. For NCD-fed mice, at the age of 16 weeks, an acute cold exposure test was performed. For HFD-fed mice, a GTT was performed at 16 weeks of age. After a 2-day acclimation period, mice were euthanized for histological and molecular analyses.

Whole-genome microarray. Microarray was performed to identify differentially expressed genes (DEGs) in brown adipose tissues between the control and obese mice. BATs were isolated from control mice and 16-week-old *ob/ob* mice. Total RNA was isolated using TRIzol (Invitrogen). RNA quantity and quality were measured by NanoDrop ND-1000. RNA integrity was determined by standard denaturing agarose gel electrophoresis. Sample labeling and array hybridization were performed according to the Agilent One-Color Microarray-Based Gene Expression Analysis protocol (Agilent Technology). The microarray profiling has been uploaded to the Gene Expression Omnibus (GEO) data repository (http://www.ncbi.nlm.nih.gov/projects/geo/) under accession number GSE191009.

Bioinformatic analysis. Agilent Feature Extraction software (version 11.0.1.1) was used to analyze acquired array images. Quantile normalization and subsequent data processing were performed using the GeneSpring GX v12.1 software package (Agilent Technologies). After quantile normalization of the raw data, genes that at least 3 out of 6 samples have flags in Detected ("All Targets Value") were chosen for further data analysis. Volcano Plot filtering identified differentially expressed genes with statistical significance between the two groups. Fold Change filtering identified differentially expressed genes (adjusted P-value < 0.05 and fold change > 1). Hierarchical clustering was performed using the R scripts. Enriched signaling pathways were identified through KEGG pathway analysis using the standard enrichment computation method.

Statistics. Data were analyzed using GraphPad Prism Software 8.0 (Graph-pad). All quantitative data are expressed as mean ± standard error of the mean (SEM). Two-group comparisons were carried out using unpaired two-tailed Student's t-test. Multi-group comparisons were carried out using one-way ANOVA with Tukey's post hoc tests. When both genotypes and treatments were considered, comparisons were carried out using two-way ANOVA with Bonferroni post hoc tests. No statistical method was used to predetermine the sample size. Sample distribution was evaluated by the Kolmogorov–Smirnov test. Non-normal data were evaluated by Spearman's rank-order correlation test. $P < 0.05$ was considered statistically significant. For cultured cell studies, data are representative of three independent experiments.

Online Supplemental material. Fig. S1 shows TRX2 deficiency in brown adipose tissue induces steatotic hypertrophy but does not significantly alter whole-body metabolism under a chow diet. Fig. S2 shows TRX2 deficiency in BAT impairs its own glucose utilization while enhances glucose metabolization in WAT, liver, and skeletal muscle. Fig. S3 shows BAT from obese mice reduced TRX2 expression and decreased FA oxidation. Fig. S4 shows characterization of brown adipocyte *in vitro* differentiation. Fig. S5 shows TRX2 loss driven NLRP3 inflammasome activation relies on mtROS in BAT. Fig. S6 shows TRX2 deficiency in BAT promotes WAT browning with no mitochondrial damage. Fig. S7 shows bocking inflammasome activity by MCC960 could not further enhance CL-induced transient increases in body temperature. Table S1 shows primer sequences used for real-time PCR analysis. Table S2 shows antibodies used for immunostaining and immunoblotting. Table S3 shows chemicals, cytokines, and other reagents.

Study approval. All animal procedures were approved by the Yale University Animal Care and Use Committee.

SUPPLEMENTAL FIGURES

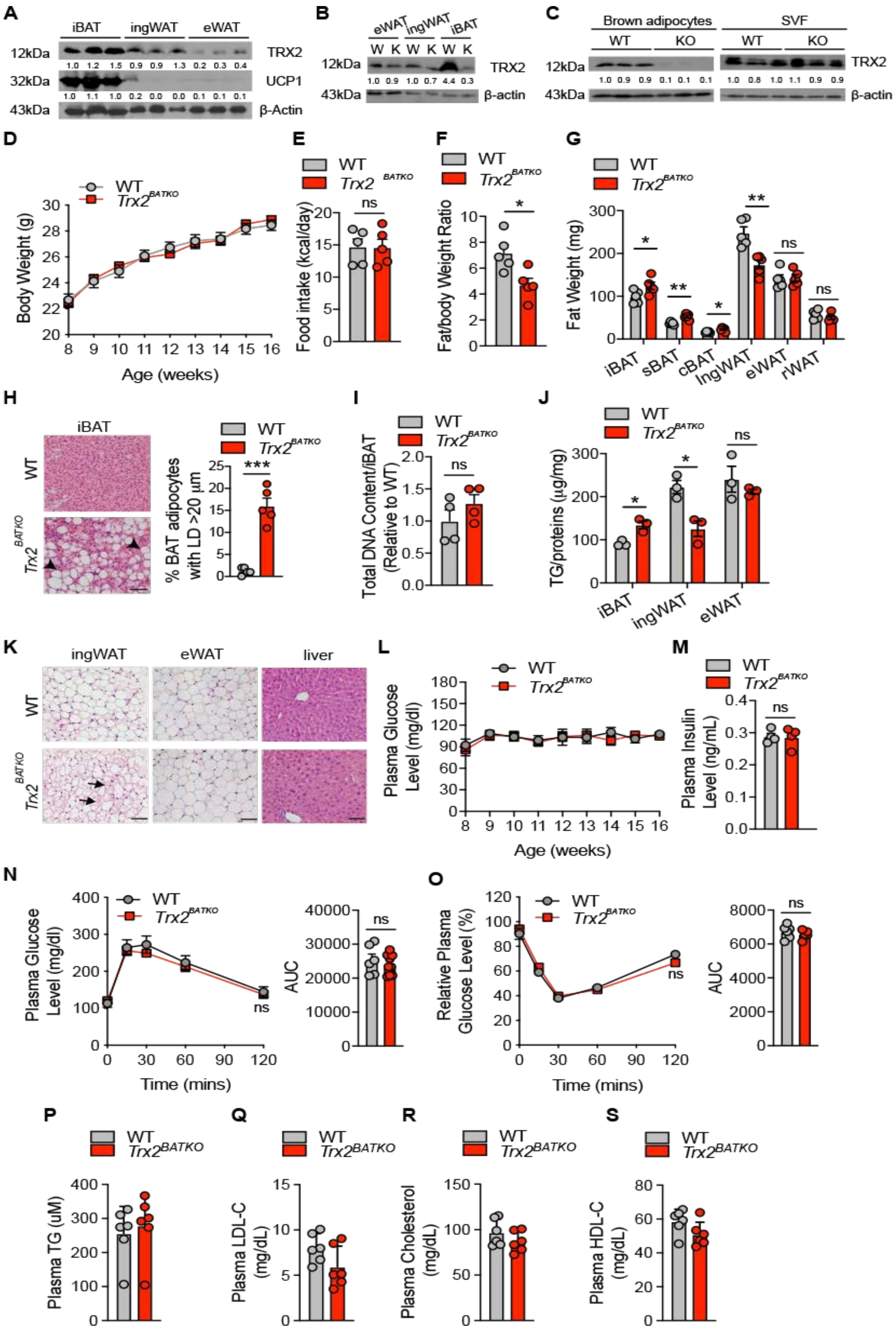

Fig. S1. TRX2 loss in brown adipose tissue induces steatotic hypertrophy but does not significantly alter whole-body metabolism under a chow diet. (A) Protein expressions of TRX2 and UCP1 from different adipose tissues from C57BL/6 mice. N=3 mice/group. (B) TRX2 protein expression in various adipose tissues from 8-week-old *Trx2*^{BATKO} and wild-type (WT) littermates (each lane contains the tissues mix from 3 mice). (C) TRX2 protein expression in purified mature brown adipocytes and stromal vascular fraction (SVF) from iBAT. (D) Body weight of *Trx2*^{BATKO} and WT mice under normal chow diet (NCD) at various ages (n=10 per group). (E) Food intake of 16-week-old *Trx2*^{BATKO} and WT mice under NCD (n=5 per group). (F) EncoMRI analysis of body composition of 16-week-old *Trx2*^{BATKO} (n=4) and WT (n=5) mice. (G) Fat mass of adipose depots from 16-week-old NCD mice (n=6 per group). (H) Representative histologic images of iBAT from 16-week-old WT and *Trx2*^{BATKO} mice. Arrowheads denote large unilocular LDs. BAT adipocytes with unilocular LD diameter≥20 μm were quantified. n=5. (I) Total DNA content in adipose depots from 16-week-old NCD mice (n=4 per group). (J) Triglyceride (TG) contents of adipose depots from 16-week-old NCD-fed *Trx2*^{BATKO} and WT mice (n=3 per group). (K) Representative histologic images of WAT and liver tissues of 16-week-old mice under NCD. Arrows denote WAT adipocytes with multilocular LDs. (L) Fasting blood glucose level of mice under NCD (n=6 per group). (M) Fasting plasma insulin level of 16-week-old NCD-fed *Trx2*^{BATKO} and WT mice (n=4 per group). (N) Glucose tolerance test of NCD treated 16-week-old mice (n=6 per group). (O) Insulin tolerance test of NCD treated 16-week-old mice (n=6 per group). (P-S) Circulating lipids, including plasma TG (P), LDL-C (R), total cholesterol (S), and HDL-C (T) level from 16-week-old WT and *Trx2*^{BATKO} mice treated with NCD (n=6/group). Quantitative data are presented as mean ± SEM. **P* < 0.05, ***P* < 0.01, and ****P* < 0.001. ns: non-significant. Significance was assessed by two-way ANOVA with Bonferroni post hoc tests (D, L, N, O), and two-tailed Student's *t*-test (E, F, G, H, I, J, M, P,Q,R,S). Scale bars: 100 μm (H, K).

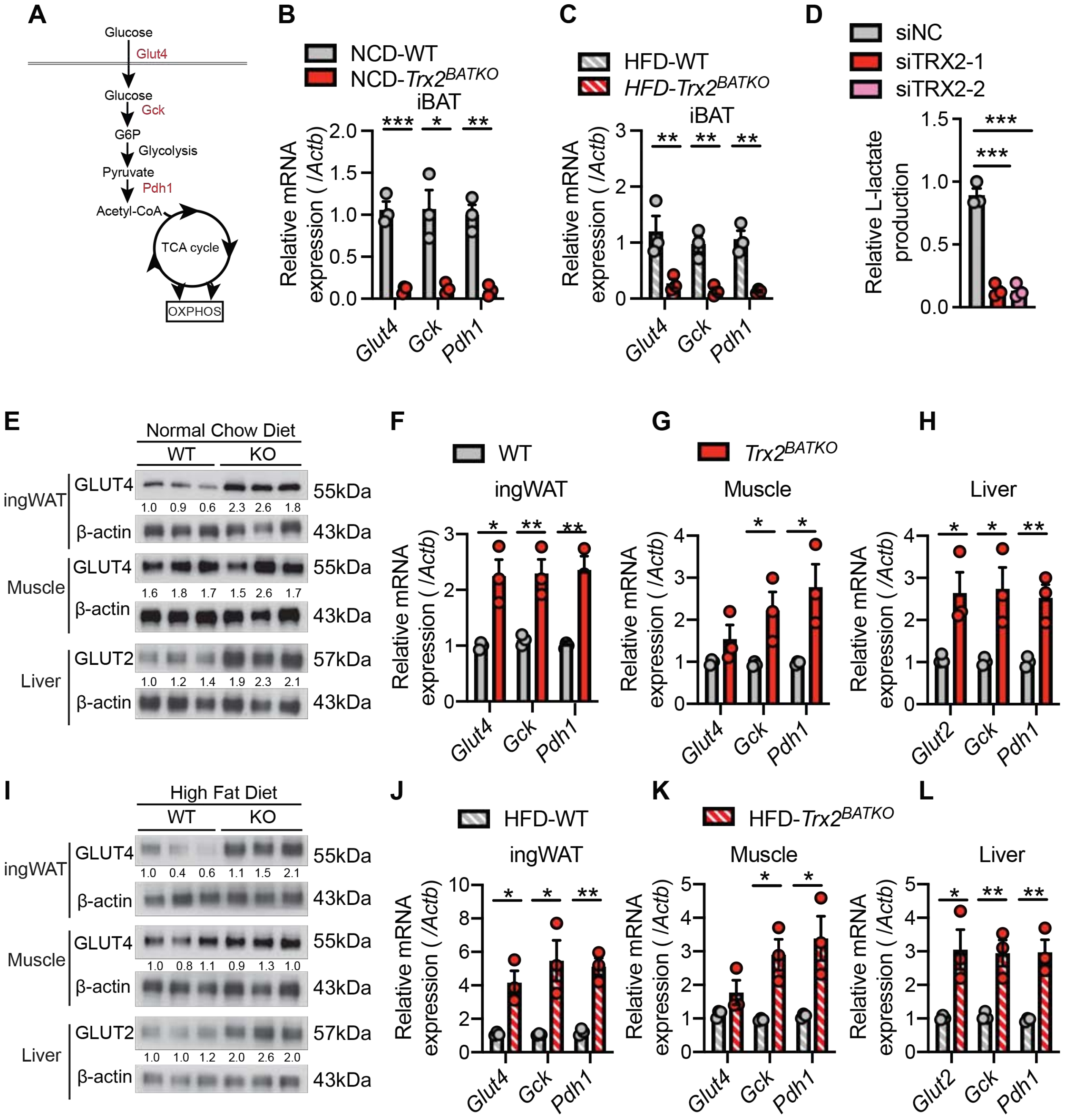

A
Glucose
Glut4
Glucose
Gck
G6P
Glycolysis
Pyruvate
Pdh1
Acetyl-CoA
TCA cycle
OXPHOS
B
NCD-WT
NCD-Trx2BATKO
iBAT
Relative mRNA expression (/Actb)
Glut4 Gck Pdh1
C
HFD-WT
HFD-Trx2BATKO
iBAT
Relative mRNA expression (/Actb)
Glut4 Gck Pdh1
D
siNC
siTRX2-1
siTRX2-2
Relative L-lactate production
E
Normal Chow Diet
WT KO
ingWAT GLUT4 55kDa
1.0 0.9 0.6 2.3 2.6 1.8
β-actin 43kDa
Muscle GLUT4 55kDa
1.6 1.8 1.7 1.5 2.6 1.7
β-actin 43kDa
Liver GLUT2 57kDa
1.0 1.2 1.4 1.9 2.3 2.1
β-actin 43kDa
F
WT
ingWAT
G
Trx2BATKO
Muscle
H
Liver
Relative mRNA expression (/Actb)
Glut4 Gck Pdh1
Glut2 Gck Pdh1
I
High Fat Diet
WT KO
ingWAT GLUT4 55kDa
1.0 0.4 0.6 1.1 1.5 2.1
β-actin 43kDa
Muscle GLUT4 55kDa
1.0 0.8 1.1 0.9 1.3 1.0
β-actin 43kDa
Liver GLUT2 57kDa
1.0 1.0 1.2 2.0 2.6 2.0
β-actin 43kDa
J
HFD-WT
ingWAT
K
HFD-Trx2BATKO
Muscle
L
Liver
Relative mRNA expression (/Actb)
Glut4 Gck Pdh1
Glut2 Gck Pdh1

Fig. S2. Deficiency of TRX2 in BAT impairs its own glucose utilization while enhances glucose metabolization in WAT, liver, and skeletal muscle. (A) The overall pathway of glucose aerobic oxidation. (B-C) Relative mRNA expression of genes involving in glucose oxidation from iBAT under NCD (B) and HFD (C) (n=3 per group). (D) In vitro glycolysis assay of differentiating 3T3-L1 brown adipocytes transfected with negative control (siNC) or *Trx2* siRNA-1 (si*Trx2*-1) or *Trx2* siRNA-2 (si*Trx2*-2) (n=3/group). (E) GLUT4 and GLUT2 proteins expression in various tissues from 16-week-old WT and $Trx2^{BATKO}$ mice with chow diet. Relative protein levels are presented by taking WT as 1.0. n=3 mice per group. (F-H) Relative mRNA expression of genes involving in glucose oxidation from ingWAT, liver and skeletal muscle fed with chow diet (n=3 per group). (I) GLUT4 and GLUT2 proteins expression in various tissues from 16-week-old WT and $Trx2^{BATKO}$ mice under HFD. Relative protein levels are presented by taking WT as 1.0. n=3 mice per group. (J-L) Relative mRNA expression of genes involving in glucose oxidation from different tissues of HFD-mice (n=3 per group). Quantitative data are presented as mean ± SEM. *$P < 0.05$, **$P < 0.01$, and ***$P < 0.001$. ns: non-significant. Significance was assessed by two-tailed Student's t-test (B,C,D,F,G,H,J,K,L).

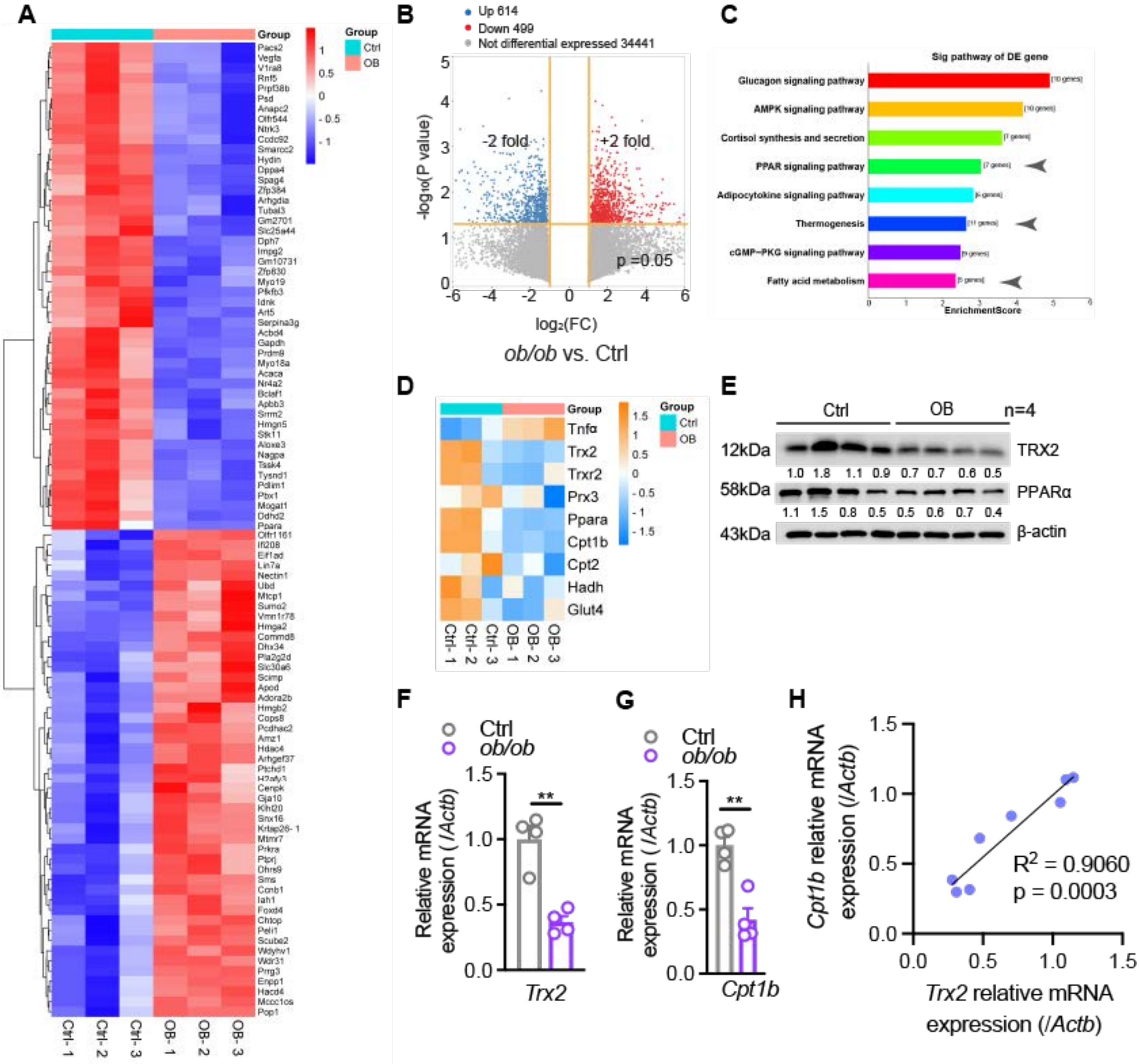

Fig. S3. BAT from obese mice show reduced TRX2 expression and decreased FA oxidation. (A) Heatmap visualization of differentially expressed genes in BAT isolated from controls (Ctrl) versus 16-week-old *ob/ob* (OB) mice (n=3/group). (B) Volcano plot showing the gene expression analysis results for the whole genome microarray; microarray indicates 614 up-regulated genes and 499 down-regulated genes with significantly different expressions in *ob/ob* mice compared to controls. (C) KEGG pathway analysis the pathways that are down-regulated in BAT of *ob/ob* mice. (D) Heat map to identify the expression of key inflammation and metabolism-related genes in BAT between the control and *ob/ob* mice. (E) Western blot analysis of TRX2 and PPARα in BAT of control and *ob/ob* mice. Protein levels were normalized by taking WT as 1.0 (n=4/ group). (F-G) *Trx2* (F) and *Cpt1b* (G) mRNA levels in BAT of control and *ob/ob* mice (n=4/ group). (H)

Correlation between the mRNA levels of *Trx2* and *Cpt1b* in BAT of control and *ob/ob* mice (n=4/ group). Quantitative data are presented as mean ± SEM. **, $P < 0.01$ versus corresponding control. Significance was assessed by two-tailed Student's t-tests (F,G). Associations were evaluated using Pearson correlation coefficients (H). BAT, brown adipose tissues.

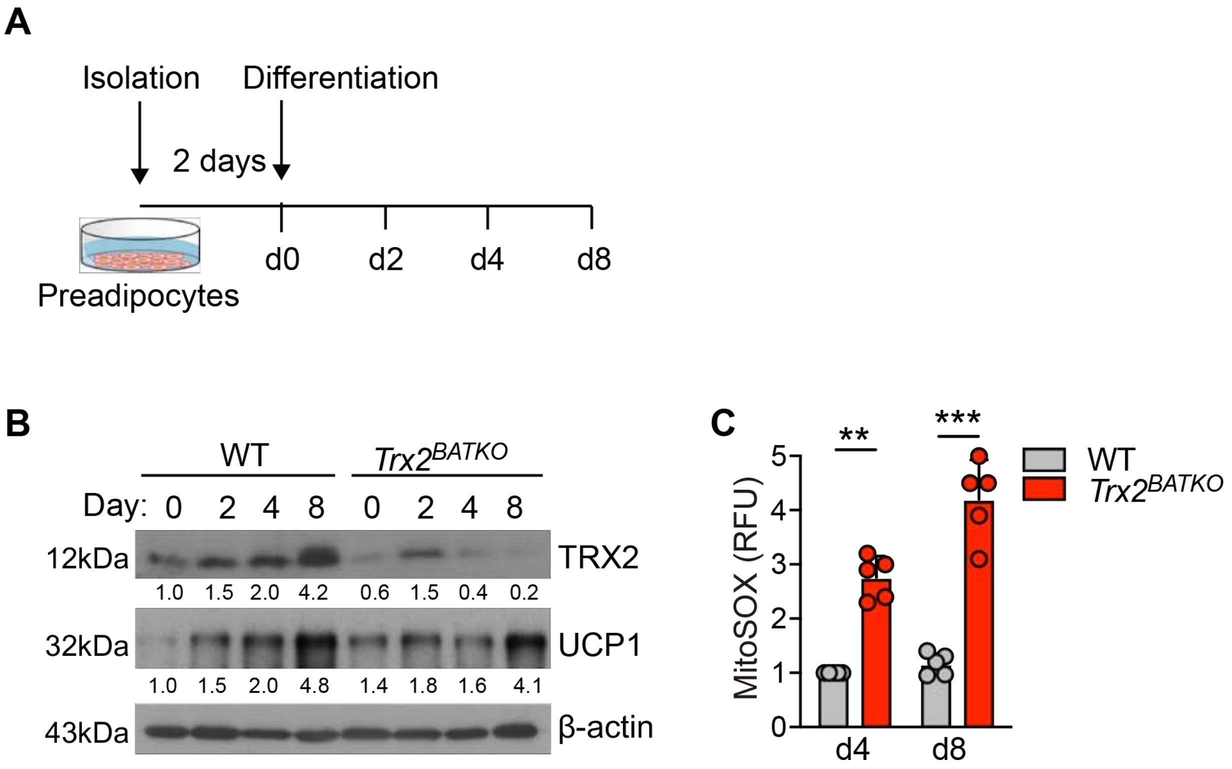

Fig. S4. Characterization of brown adipocyte in vitro differentiation. (A) A diagram for in vitro differentiation Isolated preadipocytes from WT (*Trx2*$^{lox/lox}$) and *Trx2*$^{lox/lox}$; *Ucp1*-Cre mice were cultured for 2 days to confluency and were then subjected to differentiation procedure for 0-8 days till fully differentiated (see Methods for details). (B) Cells were harvested at different time points during differentiation (day 0, 2, 4, or 8), and TRX2 and UCP1 expression were determined by Western blotting. Protein levels are presented as fold changes by taking 0 point as 1.0. Note that the *Ucp1*-Cre was expressed in mature brown adipocytes from *Trx2*$^{lox/lox}$; *Ucp1*-Cre (*Trx2*^{BATKO}) mice therefore the *Trx2* gene was specifically deleted in these cells but not in mature brown adipocytes from WT (*Trx2*$^{lox/lox}$) mice. (C) mtROS were measured by MitoSOX in day 4 (d4) and day 8 (d8)-differentiated brown adipocytes. Relative fluorescence unit are presented by taking WT as 1.0. All experiments were repeated three times (n=3). Quantitative data are presented as mean ± SEM. $^{**}P < 0.01$, and $^{***}P < 0.001$. Significance was assessed by two-tailed Student's *t*-test (C).

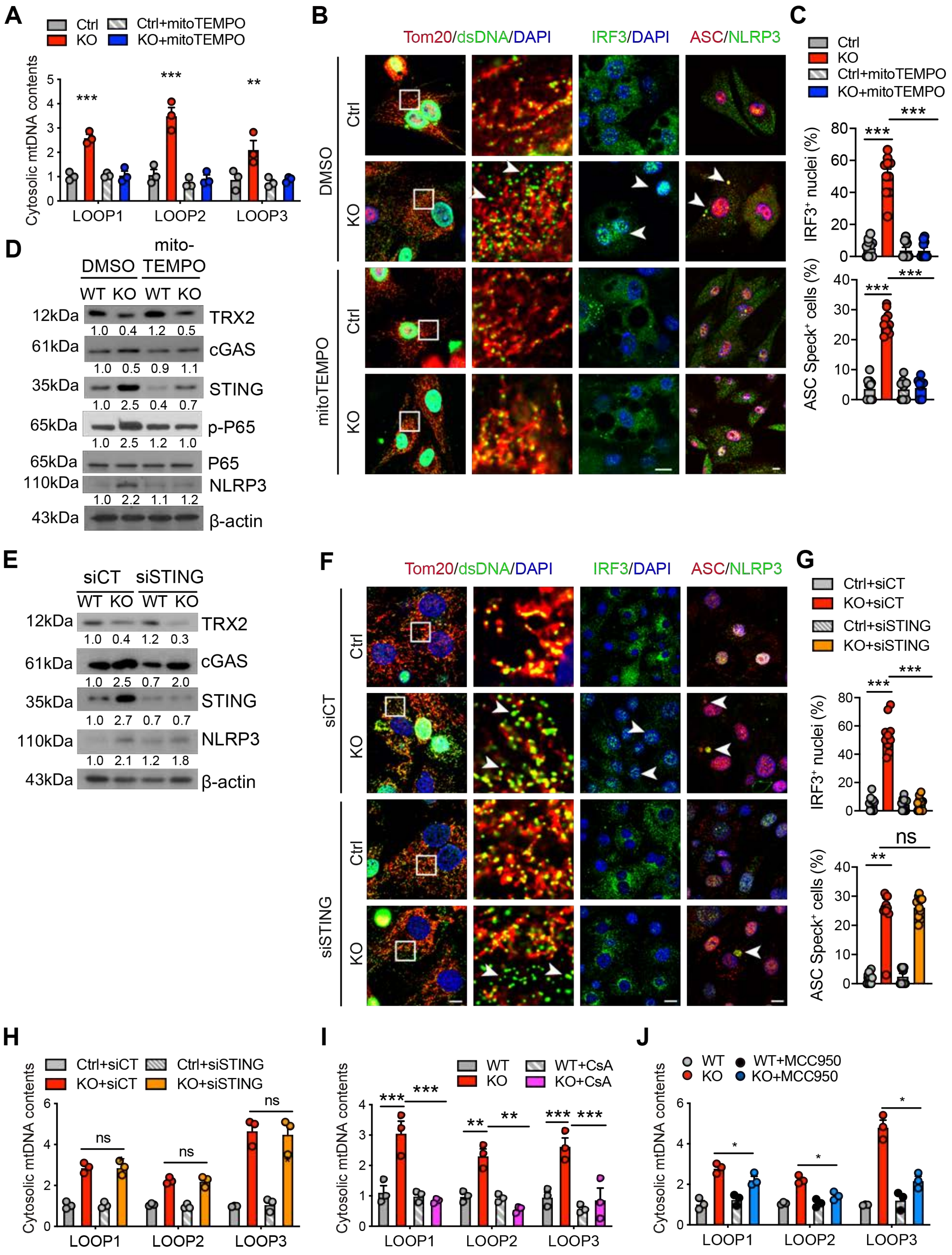

A
Ctrl
KO
Ctrl+mitoTEMPO
KO+mitoTEMPO
Cytosolic mtDNA contents
LOOP1
LOOP2
LOOP3
B
Tom20/dsDNA/DAPI
IRF3/DAPI
ASC/NLRP3
DMSO
mitoTEMPO
Ctrl
KO
C
IRF3+ nuclei (%)
ASC Speck+ cells (%)
D
DMSO
mito-TEMPO
WT KO WT KO
12kDa TRX2
1.0 0.4 1.2 0.5
61kDa cGAS
1.0 0.5 0.9 1.1
35kDa STING
1.0 2.5 0.4 0.7
65kDa p-P65
1.0 2.5 1.2 1.0
65kDa P65
110kDa NLRP3
1.0 2.2 1.1 1.2
43kDa β-actin
E
siCT
siSTING
WT KO WT KO
12kDa TRX2
1.0 0.4 1.2 0.3
61kDa cGAS
1.0 2.5 0.7 2.0
35kDa STING
1.0 2.7 0.7 0.7
110kDa NLRP3
1.0 2.1 1.2 1.8
43kDa β-actin
F
Tom20/dsDNA/DAPI
IRF3/DAPI
ASC/NLRP3
siCT
siSTING
Ctrl
KO
G
Ctrl+siCT
KO+siCT
Ctrl+siSTING
KO+siSTING
IRF3+ nuclei (%)
ASC Speck+ cells (%)
ns
H
Ctrl+siCT
KO+siCT
Ctrl+siSTING
KO+siSTING
Cytosolic mtDNA contents
LOOP1
LOOP2
LOOP3
I
WT
KO
WT+CsA
KO+CsA
Cytosolic mtDNA contents
LOOP1
LOOP2
LOOP3
J
WT
KO
WT+MCC950
KO+MCC950
Cytosolic mtDNA contents
LOOP1
LOOP2
LOOP3

Fig. S5. TRX2 loss-driven NLRP3 inflammasome activation relies on mtROS in BAT. Primary brown adipocytes were differentiated and cultured in the absence or presence of indicated inhibitors or siRNAs for 4 days. (A-D) Effects of mitoTEMPO on mtDNA release and activation of the cGAS-STING and NLRP3 inflammasome pathways. WT and KO primary brown adipocytes were treated with mitoTEMPO or vehicle (DMSO). (A) Cytosolic mtDNA contents were determined by PCR with 3 sets of mt-specific primers. Relative mtDNA contents are presented as fold changes by taking WT as 1.0 (n=3/group). (B) Immunostaining for cytosolic dsDNA (with mitochondrial marker Tom20), nuclear IRF3 or ASC/NLRP3 specks. Boxes denote magnification and arrows for cytosolic mtDNAs, nuclear IRF3 and ASC^+ specks, respectively. (C) Nuclear IRF3 translocation (% $IRF3^+$ nuclei) and % ASC $speck^+$ cells were quantified. n=10 random fields per group. (D) Western blots for the cGAS-STING and NLRP3 inflammasome pathways. Relative protein levels are presented as fold changes by taking WT as 1.0. (E-H) Effects of siSTING on mtDNA release and activation of the cGAS-STING and NLRP3 inflammasome pathways. WT and KO primary brown adipocytes were transfected with a control or STING siRNAs. (E) Western blots for the cGAS-STING and NLRP3 inflammasome pathways. Relative protein levels are presented as fold changes by taking WT as 1.0. (F) Immunostaining of cytosolic dsDNA, IRF3 translocation, and NLRP3-ASC co-localization in primary brown adipocytes treated with MCC950. Boxes denote magnification and arrows for cytosolic mtDNAs, nuclear IRF3 and ASC^+ specks, respectively. (G). Nuclear IRF3 translocation (% $IRF3^+$ nuclei) and % ASC $speck^+$ cells were quantified. n=10 random fields per group. (H-J) Effects of siSTING, CsA and MCC950 on Cytosolic mtDNA release. Cytosolic mtDNA contents were determined by qPCR with 3 sets of mt-specific primers. Relative mtDNA contents are presented as fold changes by taking WT as 1.0 (n=3/group). All experiments were repeated three times (n=3). Quantitative data are presented as mean ± SEM. $^{**}P < 0.01$, and $^{***}P < 0.001$. ns: non-significant. Significance was assessed by one-way ANOVA followed by Tukey's *post hoc* test. Scale bar: 10 μm (B, F).

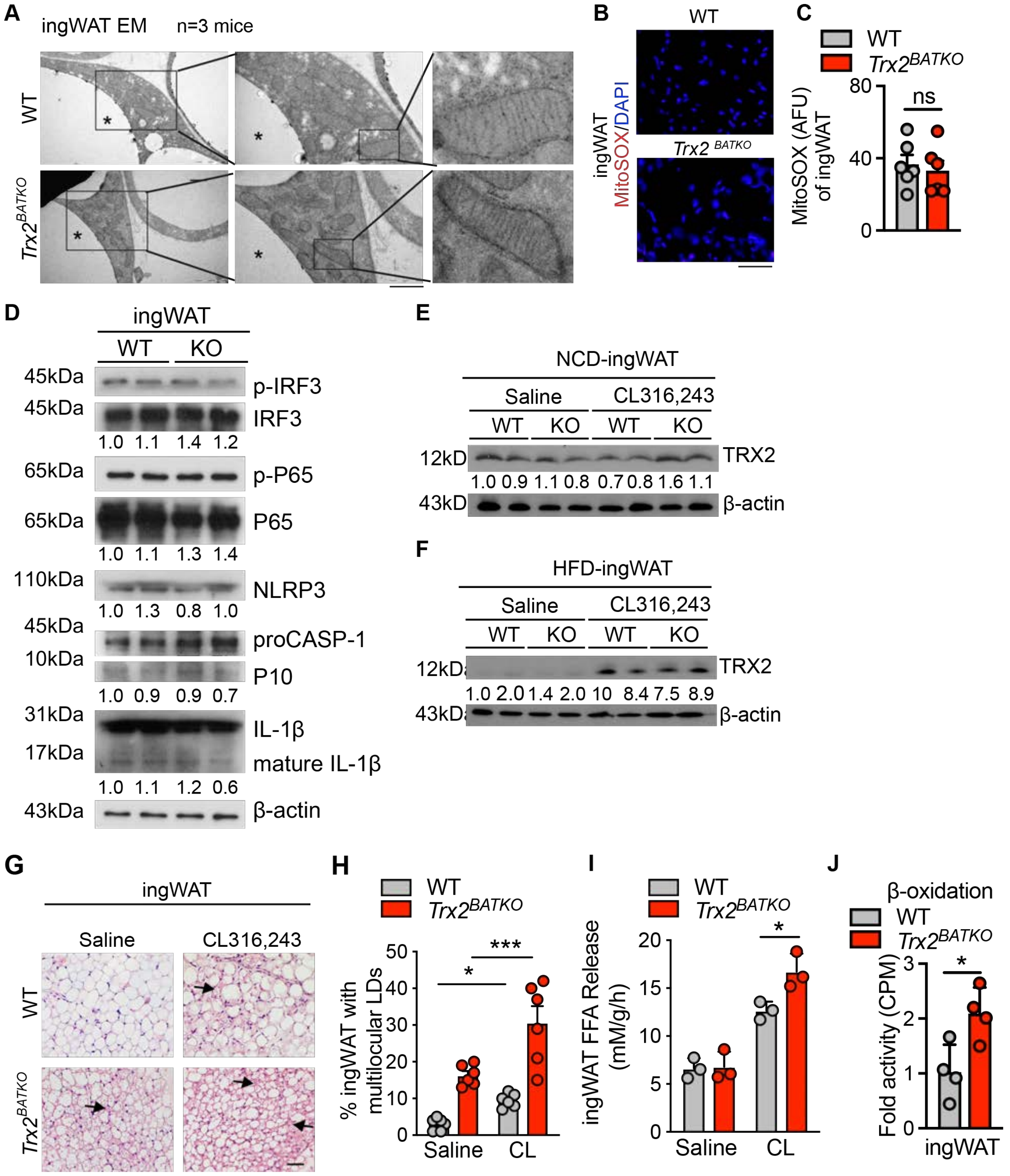

Fig. S6. TRX2 deficiency in BAT promotes ingWAT metabolism with no mitochondrial damages or inflammasome activation. (**A**) WAT tissues from 16-week-old WT and *Trx2*^{BATKO} mice were subjected to transmission electron microscopy analyses. Representative electron microscopic images (n=3/group). Boxes denote magnification and asterisks indicate LDs. (**B-C**)

Mitochondrial ROS were detected by MitoSOX (red) with DAPI counterstaining in ingWAT from 16-week-old WT and *Trx2*^{BATKO} mice. Data are presented as arbitrary fluorescence units (AFU). n=6. (**D**) Western blots of inflammasome and cGAS-cGAMP-STING pathway-related proteins from isolated ingWAT. (**E-J**) 16-week-old WT and *Trx2*^{BATKO} mice were received a single injection of CL 316,243 (CL) or saline, and ingWAT tissues were harvested at indicated times post-injection (F-H: 4 days; I-K: 3 h). (E-F) TRX2 expression of ingWAT from NCD-fed (F) or HFD-fed (G) mice treated with Saline (Basal) or CL 316,243. n=2. (G) Histologic ingWAT images. Arrows denote ingWAT with multilocular LDs. (H) % WAT adipocytes with multilocular LDs are quantified. n=6. (I) *Ex vivo* lipolysis assay by detecting free fatty acids (FFA) levels of isolated ingWAT (n=3/group). (J) *Ex vivo* fatty acid oxidation of isolated ingWAT (n=3/group). Quantitative data are presented as mean ± SEM. **P* < 0.05, ***P* < 0.01 and ****P* < 0.001. Significance was assessed by one-way ANOVA followed by Tukey's *post hoc* test. Scale bar: 1 μm (A); 100 μm (B, G).

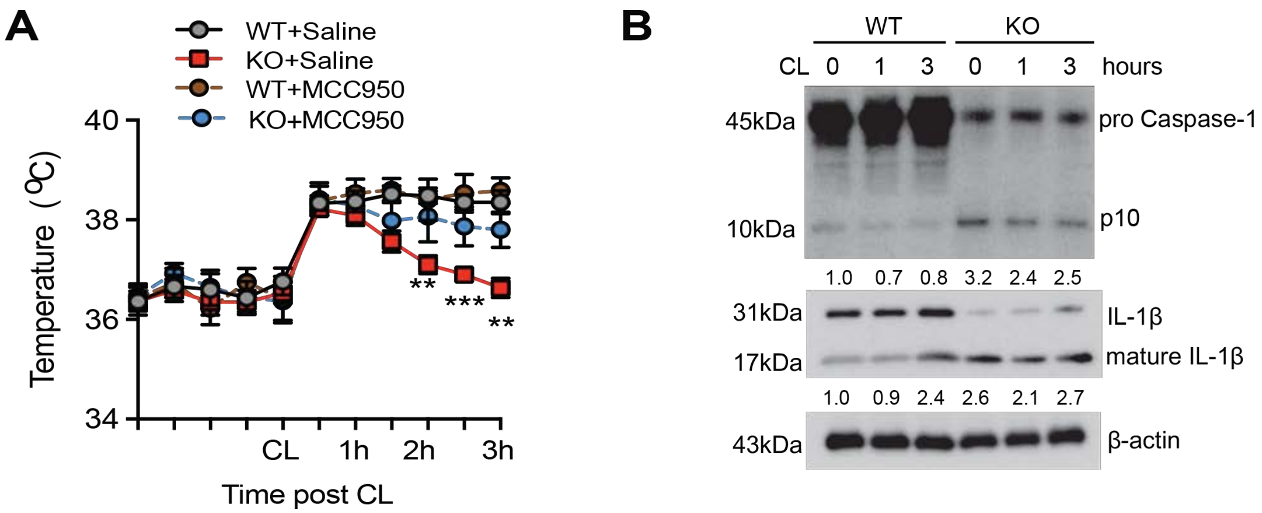

Fig. S7. Blocking inflammasome activity by MCC960 could not further enhance CL-induced transient increases in body temperature. (**A**) 8-week-old WT and *Trx2*^{BATKO} mice were treated with MCC950 (10 mg/kg) or an equal volume of saline by intraperitoneal injection every other day for 8 weeks. Mice were then treated with CL136,243 (1 mg/kg) and intrarectal temperature were measured before and after CL treatment as indicated (n=6/group). Quantitative data are presented as mean ± SEM. $^{**}P < 0.01$, and $^{***}P < 0.001$. Significance was assessed by one-way ANOVA followed by Tukey's *post hoc* test. (**B**) Immunoblots of proteins involved in inflammasome from iBAT of WT and *Trx2*^{BATKO} mice treated with CL136,243 (1mg/kg). P10 protein levels and mature IL1β/Il-1β ratios are presented as fold changes by taking saline-treated WT as 1.0. Each lane contains the tissues mixed from 3 mice.

Table S1. Primer sequences used for real-time PCR analysis.

Gene	Forward primer	Reverse primer
mtDNA loop 1	AATCTACCATCCTCCGTGAAACC	TCAGTTTAGCTACCCCCAAGTTTAA
mtDNA loop 2	CCCTTCCCCATTTGGTCT	TGGTTTCACGGAGGATGG
mtDNA loop 3	TCCTCCGTGAAACCAACAA	AGCGAGAAGAGGGGCATT
Tert	CTAGCTCATGTGTCAAGACCCTCTT	GCCAGCACGTTTCTCTCGTT
Glut4	GGCATGGGTTTCCAGTATGT	CCTCTGGTTTCAGGCACTTT
Glut2	CACATCCTACTTGGCCTATCTG	CTTTGCCCTGACTTCCTCTT
Gck	AACACAAGAAACTACCCCTGG	GGATGCCCTTGTCTATGTCTTC
Pdh1	CCACCTCATCACTGCCTATC	CCTTTAGCACAACCTCCTC TT
Fasn	CCCCTCTGTTAATTGGCTCC	TTGTGGAAGTGCAGGTTAGG
Pc	GAGCTTATCCCGAACATCCC	TCCATACCATTCTCTTTGGCC
Ppara	GACATGGAGACCTTGTGTATGG	AGGAACTCGCGTGTGATAAAG
Cpt1b	AGGCAGTAGCTTTCCAGTTC	GGAGTTGATTCCAGACAGGTAG
Cpt2	CCTGCATACCAGCGGATAAA	CCTATCCAGTCATCGTGAACAG
Acsl1	GCTGTGCACCCGGAATTA	GGTGGCGTACAGTTCATCTATC
Acadm	GAAGACGTCAGAGTGCCTAA	CGACTGTAGGTCTGGTTCTATC
Acadl	CCCATGGCATTAGCCTCTTT	GCTGTGTCCTGAGCTTTCAT
Hadh	CCAAGAAGGGAATTGAGGAGAG	TCGGTGCTGGTTGAAAGG
Angptl4	ACTTCAGATGGAGGCTGGAC	TCCGAAGCCATCCTTGTAGG
Acc1	AAGGCTATGTGAAGGATGTGG	CTGTCTGAAGAGGTTAGGGAAG
PGC1a	CGACAGCTATGAAGCCTATGAG	CTTCTGCCTCTCTCTCTGTTTG
Ucp1	CACGGGGACCTACAATGCTT	TAGGGGTCGTCCCTTTCCAA
Prdm16	CACAAGTCCTACACGCAGTT	TTGTTGAGGGAGGAGGTAGT
Dio2	GGTAGCCTTTGAACGTGTGT	CCAACTTCGGACTTCTTGTAGG
Il-6	CAAAGCCAGAGTCCTTCAGAG	GTCCTTAGCCACTCCTTCTG
Tnfa	CTTCTGTCTACTGAACTTCGGG	CAGGCTTGTCACTCGAATTTTG
Ifna	GATGCCCAGCAGATCAAGAA	CATGCAGCAGATGAGTCCTT
Ifnb	GTTGTACGTCTCCTGGATGAAC	CCTTTGCACCCTCCAGTAATAG
Nlrp3	GGAGTTCTTCGCTGCTATGT	AACAACCTCCTGGTCCTTTC
Caspase1	TACACGTCTTGCCCTCATTATC	CTCCAGCAGCAACTTCATTTC
Il1b	GGTGTGTGACGTTCCCATTA	TCCTGACCACTGTTGTTTCC
Il18	GGAGACCTGGAATCAGACAAC	CAGTCATATCCTCGAACACAGG
Actb	GTTGGTTGGAGCAAACATC	CTTATTTCATGGATACTTGGAATG

Table S2. Antibodies used for immunostaining and immunoblotting

TRX2	Abcam	Cat# ab185544;<br>RRID: AB_2737587
β-actin	Santa Cruz	Cat# sc-47778;<br>RRID: AB_2714189
PPARγ	Santa Cruz	Cat# sc-7273;<br>RRID: AB_628115
PPARα	Abcam	Cat#ab24509<br>RRID: AB_448110
HSL	Cell Signaling Technology	Cat# 4107;<br>RRID: AB_2296900
GLUT4	Santa Cruz	Cat# sc-53566;<br>RRID: AB_629533
GLUT2	Santa Cruz	Cat# sc-518022;<br>RRID: AB_2890905
UCP1	Abcam	Cat# ab10983<br>RRID: AB_2241462
LPL	Santa Cruz	Cat# sc-32885<br>RRID: AB_2234585
TRXR2	Upstate	Cat# 07-079
PRX3	Abcam	Cat# ab73349;<br>RRID: AB_1860862
GPx1	Lab Frontier	Cat# LF-PA0019
SOD1	Santa Cruz	Cat# sc-11407<br>RRID: AB_2193779
SOD2	R&D Systems	Cat# MAB3419<br>RRID: AB_2191831
TRX1	Cell Signaling Technology	Cat# 2429<br>RRID: AB_2272594
Total OXPHOS Rodent WB Antibody Cocktail	Abcam	Cat# ab110413;<br>RRID: AB_2629281
VDAC	Cell Signaling Technology	Cat# 4661;<br>RRID: AB_10557420
Histone H3	Cell Signaling Technology	Cat# 9715

		RRID: AB_331563
Calnexin	BD Transduction Laboratories	Cat#51-9001939
cGAS	Cell Signaling Technology	Cat# 31659<br>RRID: AB_2799008
STING	Cell Signaling Technology	Cat# 13647<br>RRID: AB_2732796
Phospho-IRF-3	Cell Signaling Technology	Cat# 4947<br>RRID: AB_823547
IRF-3	Cell Signaling Technology	Cat# 4302<br>RRID: AB_1904036
Phospho-TBK1/NAK	Cell Signaling Technology	Cat# 5483<br>RRID: AB_10693472
TBK1/NAK	Cell Signaling Technology	Cat# 3013<br>RRID:
Phospho-NF-κB p65	Cell Signaling Technology	Cat# 3033;<br>RRID: AB_331284
NF-κB p65	Cell Signaling Technology	Cat# 8242;<br>RRID: AB_10859369
AIM2	Cell Signaling Technology	Cat# 63660<br>RRID: AB_2199749
NLRP3	Cell Signaling Technology	Cat# 15101<br>RRID: AB_2722591
Cleaved Caspase-1	Cell Signaling Technology	Cat# 4199<br>RRID: AB_1903916
Caspase-1	Cell Signaling Technology	Cat# 3866<br>RRID: AB_2069051
IL-1β	R&D Systems	Cat# AF-401-NA<br>RRID: AB_416684
Phospho-p38 MAPK (Thr180/Tyr182)	Cell Signaling Technology	Cat# 4511<br>RRID: AB_2139682
p38α	Santa Cruz	Cat# sc-535<br>RRID: AB_632138
Phospho-SAPK/JNK	Cell Signaling Technology	Cat# 9255

		RRID: AB_2307321
JNK	Santa Cruz	Cat# sc-7345<br>RRID: AB_675864
Phospho-ASK1(Thr845)	Cell Signaling Technology	Cat# 3765<br>RRID: AB_2139929
ASK1	Cell Signaling Technology	Cat# 3762<br>RRID: AB_2139939
Caspase-3	Cell Signaling Technology	Cat# 9665;<br>RRID: AB_2069872
TOM20	Cell Signaling Technology	Cat# 42406<br>RRID: AB_2687663
dsDNA	Abcam	Cat# ab27156<br>RRID: AB_470907
NLRP3	Abcam	Cat# ab4207<br>RRID: AB_955792
ASC	AdipoGen	Cat#AG-25B-0006<br>RRID: AB_2490440
Alexa 700-CD45.2	Biolegend	Cat#109821<br>RRID: AB_493730
Alexa 488-CD4	Biolegend	Cat#100425<br>RRID: AB_ 493520
PE-F4/80	Biolegend	Cat#123109<br>RRID: AB_ 893498
APC/Cyanine7-CD8a	Biolegend	Cat#100713<br>RRID: AB_ 312752
APC-CD11b	Biolegend	Cat#101211<br>RRID: AB_ 312794
TruStain FcX™ (anti-mouse CD16/32)	Biolegend	Cat#101320<br>RRID: AB_ 1574975
FITC-Ly-6G/LY-6C	BD Biosciences	Cat#553126<br>RRID: AB_ 394642

Table S3. Chemicals, cytokines and other reagents

High-fat rodent diet	Research Diets	Cat# D12108C
VECTASHIELD MOUNTING MEDIUM with DAPI	Vector Laboratories	Cat# H-1200
BODIPY 493/503	Invitrogen	Cat# D3922
MitoSox Red	Invitrogen	Cat# M36008
MitoTracker™ Green FM	Invitrogen	Cat# M7514
JC-1 Dye	Invitrogen	Cat# T3168
Hoechst 33342	Invitrogen	Cat# H3570
TRIzol™ Reagent	Invitrogen	Cat# 15596018
MitoTEMPO	Enzo	Cat# ALX-430-150
Cyclosporin A	Sigma Aldrich	Cat# C3662
MCC950	Selleckchem	Cat# S7809
STING siRNA	Santa Cruz	Cat# sc-154411
ECL	Santa Cruz	Cat# sc-2048
Hematoxylin Solution	Sigma Aldrich	Cat# GHS216
Eosin Y solution	Sigma Aldrich	Cat# HT110116
**Critical Commercial Assays**		
Mouse Ultrasensitive Insulin ELISA	ALPCO	Cat# 80-INSMSU-E01
Free Fatty Acid Quantification Colorimetric/Fluorometric Kit	BioVision	Cat# K612
Triglyceride Assay Kit - Quantification	Abcam	Cat# ab65336
Mouse IL-1 beta ELISA Kit	Abcam	Cat# ab197742
In Situ Cell Death Detection Kit, Fluorescein	Sigma Aldrich	Cat# 11684795910

GoTaq Green Master Mix	Promega	Cat# M712B
RNeasy Mini Kit (250)	QIAGEN	Cat# 74106
DNeasy Blood & Tissue Kit	QIAGEN	Cat# 69504
iScript cDNA Synthesis Kit	BioRad	Cat# 1708890
iQ SYBR Green Supermix	BioRad	Cat# 1708882
Mitochondria Isolation Kit for Tissue	Thermo Fisher Scientific	Cat# 89801
Adenosine 5¢ -triphosphate (ATP) Bioluminescent Assay Kit	Sigma Aldrich	Cat# FLAA
Seahorse XFe96 FluxPak mini	Agilent Technologies	Cat# 102601-100
Seahorse XF Cell Mito Stress Test Kit	Agilent Technologies	Cat# 103015-100
QIAamp DNA Mini Kit(50)	QIAGEN	Cat# 51304
**Analyses tools**		
ImageJ software	NIH	RRID: SCR_003070
Seahorse Wave	Agilent Technologies	RRID: SCR_014526
Prism 8	GraphPad Software	RRID: SCR_002798

REFERENCES

1. Huang Q, Zhou HJ, Zhang H, Huang Y, Hinojosa-Kirschenbaum F, Fan P, et al. Thioredoxin-2 inhibits mitochondrial reactive oxygen species generation and apoptosis stress kinase-1 activity to maintain cardiac function. *Circulation.* 2015;131(12):1082-97.
2. Aryal B, Singh AK, Zhang X, Varela L, Rotllan N, Goedeke L, et al. Absence of ANGPTL4 in adipose tissue improves glucose tolerance and attenuates atherogenesis. *JCI Insight.* 2018;3(6).
3. Singh AK, Aryal B, Chaube B, Rotllan N, Varela L, Horvath TL, et al. Brown adipose tissue derived ANGPTL4 controls glucose and lipid metabolism and regulates thermogenesis. *Mol Metab.* 2018;11:59-69.
4. Shin H, Ma Y, Chanturiya T, Cao Q, Wang Y, Kadegowda AKG, et al. Lipolysis in Brown Adipocytes Is Not Essential for Cold-Induced Thermogenesis in Mice. *Cell Metab.* 2017;26(5):764-77 e5.
5. Bai J, Cervantes C, Liu J, He S, Zhou H, Zhang B, et al. DsbA-L prevents obesity-induced inflammation and insulin resistance by suppressing the mtDNA release-activated cGAS-cGAMP-STING pathway. *Proc Natl Acad Sci U S A.* 2017;114(46):12196-201.
6. Chu DT, Malinowska E, Gawronska-Kozak B, and Kozak LP. Expression of adipocyte biomarkers in a primary cell culture models reflects preweaning adipobiology. *J Biol Chem.* 2014;289(26):18478-88.
